# Variation in health system performance for managing diabetes among states in India: a cross-sectional study of individuals aged 15 to 49 years

**DOI:** 10.1186/s12916-019-1325-6

**Published:** 2019-05-13

**Authors:** Jonas Prenissl, Lindsay M. Jaacks, Viswanathan Mohan, Jennifer Manne-Goehler, Justine I. Davies, Ashish Awasthi, Anne Christine Bischops, Rifat Atun, Till Bärnighausen, Sebastian Vollmer, Pascal Geldsetzer

**Affiliations:** 1 Heidelberg Institute of Global Health, Heidelberg University, Im Neuenheimer Feld 130/3, 69120 Heidelberg, Germany; 2 Medical Faculty Mannheim, Heidelberg University, Mannheim, Germany; 3 Department of Global Health and Population, Harvard T.H. Chan School of Public Health, Boston, MA USA; 4 Public Health Foundation of India, New Delhi, Delhi NCR India; 5 Madras Diabetes Research Foundation & Dr. Mohan’s Diabetes Specialities Centre, Chennai, Tamil Nadu India; 6 Division of Infectious Diseases, Massachusetts General Hospital, Harvard Medical School, Boston, MA USA; 7 MRC/Wits Rural Public Health and Health Transitions Research Unit, School of Public Health, Education Campus, University of Witwatersrand, Johannesburg, Gauteng South Africa; 8 Institute of Applied Health Research, University of Birmingham, Birmingham, UK; 9 Indian Institute of Public Health, Gandhinagar, Gujarat India; 10 Harvard Medical School, Harvard University, Boston, MA USA; 11 Africa Health Research Institute, Mtubatuba, KwaZulu-Natal South Africa; 12 Department of Economics and Centre for Modern Indian Studies, University of Goettingen, Göttingen, Germany

**Keywords:** Diabetes, India, Care cascade, Health system performance

## Abstract

**Background:**

Understanding where adults with diabetes in India are lost in the diabetes care cascade is essential for the design of targeted health interventions and to monitor progress in health system performance for managing diabetes over time. This study aimed to determine (i) the proportion of adults with diabetes in India who have reached each step of the care cascade and (ii) the variation of these cascade indicators among states and socio-demographic groups.

**Methods:**

We used data from a population-based household survey carried out in 2015 and 2016 among women and men aged 15–49 years in all states of India. Diabetes was defined as a random blood glucose (RBG) ≥ 200 mg/dL or reporting to have diabetes. The care cascade—constructed among those with diabetes—consisted of the proportion who (i) reported having diabetes (“aware”), (ii) had sought treatment (“treated”), and (iii) had sought treatment and had a RBG < 200 mg/dL (“controlled”). The care cascade was disaggregated by state, rural-urban location, age, sex, household wealth quintile, education, and marital status.

**Results:**

This analysis included 729,829 participants. Among those with diabetes (19,453 participants), 52.5% (95% CI, 50.6–54.4%) were “aware”, 40.5% (95% CI, 38.6–42.3%) “treated”, and 24.8% (95% CI, 23.1–26.4%) “controlled”. Living in a rural area, male sex, less household wealth, and lower education were associated with worse care cascade indicators. Adults with untreated diabetes constituted the highest percentage of the adult population (irrespective of diabetes status) aged 15 to 49 years in Goa (4.2%; 95% CI, 3.2–5.2%) and Tamil Nadu (3.8%; 95% CI, 3.4–4.1%). The highest absolute number of adults with untreated diabetes lived in Tamil Nadu (1,670,035; 95% CI, 1,519,130–1,812,278) and Uttar Pradesh (1,506,638; 95% CI, 1,419,466–1,589,832).

**Conclusions:**

There are large losses to diabetes care at each step of the care cascade in India, with the greatest loss occurring at the awareness stage. While health system performance for managing diabetes varies greatly among India’s states, improvements are particularly needed for rural areas, those with less household wealth and education, and men. Although such improvements will likely have the greatest benefits for population health in Goa and Tamil Nadu, large states with a low diabetes prevalence but a high absolute number of adults with untreated diabetes, such as Uttar Pradesh, should not be neglected.

**Electronic supplementary material:**

The online version of this article (10.1186/s12916-019-1325-6) contains supplementary material, which is available to authorized users.

## Introduction

India—home to over one sixth of the world’s population [1]—is in the midst of a diabetes epidemic [2, 3]. In nationally representative studies of India from 2012 to 2014, we recently reported a crude prevalence of diabetes of 7.5% (95% CI, 7.3–7.7%) [4], as well as a high predicted cardiovascular disease (CVD) risk across all population groups [5]. The United Nations (UN) member states agreed to reduce premature mortality from non-communicable diseases (NCDs) by one third by 2030 (Sustainable Development Goal [SDG] target 3.4), and the World Health Organization (WHO) member states to halt the rise of diabetes by 2025 [6, 7]. Failing to meet these targets will result in high avoidable morbidity and mortality and a substantial economic burden from lost productivity and increased healthcare costs [8].

Strong health system performance for diabetes across the care continuum—from screening and early detection to timely treatment and long-term adherence—is essential to achieve glycemic control and prevent complications of diabetes [9, 10]. In addition to providing a benchmark for future comparison, understanding the current state of health system performance for diabetes in India could directly inform the design of targeted interventions and programs that give patients the best chance of achieving good glycemic control. A useful approach to studying health system performance in managing chronic diseases is the cascade of care. Initially conceptualized to monitor human immunodeficiency virus (HIV) program effectiveness [11], and then to examine achievement of global HIV care goals [12], the care cascade approach has recently been applied to examine the management of diabetes in Malawi [13], South Africa [14], and the USA [15]. The care cascade is based on the idea that those with a particular chronic condition transition across a number of care steps (e.g., screening, diagnosis, and initiation of treatment) before they can achieve successful “control” of the condition. Apart from its simplicity and relation to the care process in a program, and hence interpretability by policy makers and practitioners, a key advantage of this approach is that it clearly depicts along which steps patients are “lost” to management across the care continuum in a health system.

While we have examined health system performance for the management of diabetes in 12 countries in sub-Saharan Africa [16], no such study exists for India. The Indian Council of Medical Research—India Diabetes (ICMR-INDIAB) study provided estimates of diabetes awareness among 57,000 adults in 14 of 29 states and one of seven Union Territories of India and glycemic control estimates among a smaller subset of 14,200 adults [17, 18]. This study, however, is the first to use large-scale population-based data from all states and Union Territories to comprehensively assess health system performance for diabetes in India and its variation among states. Specifically, analyzing data from 730,000 adults with a random plasma glucose measurement, we aimed to assess (i) the proportion of adults with diabetes in India who have reached each step of the diabetes care cascade (awareness of diagnosis, sought treatment, and—with some important limitations—glycemic control) and (ii) the variation of these care cascade indicators among states and socio-demographic groups across the country.

## Methods

### Data source

We used data from the fourth National Family Health Survey (NFHS-4), which was carried out between 2015 and 2016 and covered all districts (using district delineations from the time of the 2011 India census) [19] in the 29 states and seven Union Territories (henceforth also referred to as “states”) of India. Within each district, the sampling process was carried out differently in rural than in urban areas. In rural areas, villages were used as primary sampling units (PSUs), which were selected with probability proportional to size, while census enumeration blocks—selected through simple random sampling—were used as PSUs in urban areas. Twenty-two households in each PSU were selected through systematic random sampling after a complete mapping and household listing in the selected PSUs. Within all selected households, all non-pregnant women aged 15–49 years who had stayed in the household the night prior to the survey (including both usual residents and visitors) were eligible for the survey questionnaire and a capillary blood glucose measurement. Men aged 15–54 years in a household were only eligible in a random subsample of 15% of households. We excluded men aged 50–54 years from this survey to have consistent age ranges among men and women. The response rate among eligible women and men was 96.7% and 91.9%, respectively. More details about the methodology of the NFHS-4 can be found in Additional file 1: Methods S1.

### Ethics

This analysis of an existing data set in the public domain received a determination of “not human subjects research” by the institutional review board of the Harvard T. H. Chan School of Public Health on 9 May 2018.

### Ascertaining diabetes

All participants were requested to undergo a one-time capillary blood glucose measurement using a handheld blood glucometer (FreeStyle Optium H, manufactured by Abbott Laboratories). Whole blood glucose measures were converted into plasma-equivalent blood glucose by multiplying with 1.11 [20]. Fasting was defined as reporting to have neither eaten nor drunk anything besides water for at least 12 h. Participants were not instructed to fast prior to the blood glucose measurement. Only 1.1% of participants reported to be fasted at the time of the measurement. Individuals with missing information on whether or not they had fasted (0.3% of participants after excluding individuals with a missing blood glucose measurement) were excluded from this analysis. Diabetes was defined as having responded with “yes” to the question “Do you currently have diabetes?” or having a high plasma-equivalent blood glucose reading (≥ 200 mg/dL [11.1 mmol/L] if participants reported not to have fasted or ≥ 126 mg/dL [7.0 mmol/L] if participants reported to be fasted).

### Ascertaining the diabetes care cascade

The diabetes care cascade was constructed among only those who had diabetes as per the definition above. “Aware” was defined as having responded with “yes” to the question “Do you currently have diabetes?” Only those who were “aware” were asked whether they had sought treatment. “Treated” was defined as having responded with “yes” to the question “Have you sought treatment for this issue [diabetes]?” Due to a lack of glycated hemoglobin A1c data (HbA1c), “controlled” was defined as being “treated” and having a plasma-equivalent blood glucose below the threshold for diabetes used in this study (< 200 mg/dL if not fasted and < 126 mg/dL if fasted). Among urban African-Americans in the USA, this threshold was found to have a positive predictive value for a HbA1c > 8.0% of between 80 and 85% [21]. While treatment guidelines generally consider a lower HbA1c cutoff to indicate good glycemic control [22], we chose this somewhat higher threshold to obtain a more conservative measure of being “uncontrolled.” The rationale for preferring a conservative measure is that policy makers may only want to endeavor to improve health system performance for diabetes if they can be reasonably certain that the system’s performance is insufficient. We thus favored misclassifying an “uncontrolled” individual as being “controlled” rather than the reverse. The outcomes “unaware,” “untreated,” and “uncontrolled” were defined as the reciprocal values of “aware,” “treated,” and “controlled” among those who had diabetes. The calculation of state population estimates of these unmet needs for care variables is described in Additional file 1: Methods S2.

### Socio-demographic variables

The socio-demographic variables used as independent variables in this analysis were state, household wealth index quintile, educational attainment, marital status (currently married or not), and whether the household was located in a rural or urban area. The household wealth index was created based on a principal component analysis of binary variables indicating household characteristics and household ownership of durable goods as described in more detail in Additional file 1: Methods S3. Education was categorized as “Primary school or less,” “Secondary school unfinished,” and “Secondary school or above.” The category “Primary school or less” includes individuals without any formal education, individuals who went to primary school but did not finish, and individuals who completed primary school.

### Statistical analysis

We constructed the diabetes care cascade for the national sample (total, by sex, and rural versus urban areas) and disaggregated by state using sampling weights to account for the survey design. The sampling weights were also adjusted for the higher probability of sampling women than men. To investigate the association between diabetes prevalence and care cascade indicators (i.e., the proportion of those with diabetes who reached each step of the care cascade) in a state/district, we plotted diabetes prevalence against the care cascade indicator for each state and each district. Lastly, we used covariate-adjusted logistic regression models to investigate the association of care cascade indicators with individual-level socio-demographic characteristics. These regressions included a binary indicator (“fixed effect”) for each of 640 districts to filter out effects associated with the participants’ district on the outcomes. We used restricted cubic splines with five knots for age in all regressions to avoid loss of information due to categorization of this continuous variable. The knots were placed at the 5th, 27.5th, 50th, 72.5th, and 95th percentiles of the age distribution.

Socio-demographic information was available on 749,119 participants (647,451 women and 101,668 men) when excluding pregnant women. 2.6% (19,290/749,119) of all participants had a missing blood glucose measurement or missing information on whether or not they had fasted and were thus excluded, resulting in a sample of 729,829 participants (631,825 women and 98,004 men) for analysis. All analyses were complete case analyses. Statistical analyses were performed in R version 3.3.2.

## Results

### Sample characteristics

Individuals with a missing blood glucose measurement or fasting status—2.6% (19,290/749,119) of all participants with socio-demographic information—were more likely to live in an urban area, have a higher educational attainment, live in a wealthier household, and be men than those with non-missing values (Additional file 1: Table S1). Table 1 shows the (unweighted) characteristics of the included participants. 49.5% of participants were younger than 30 years, 40.3% went to secondary school but did not complete secondary school, 68.7% were married, and 29.5% lived in urban areas. 2.7% of the participants had diabetes. There were no missing values for age, education, household wealth index, marital status, or urban area.

**Table 1:** Sample characteristics

Characteristic	Total	Female	Male
No.	729,829	631,825	98,004
Diabetes, no. (%)	19,453 (2.7)	16,260 (2.6)	3193 (3.3)
Fasted, no. (%)	8317 (1.1)	7242 (1.1)	1075 (1.1)
Age group, no. in years (%)			
15–19	131,984 (18.1)	113,974 (18.0)	18,010 (18.4)
20–24	116,099 (15.9)	100,551 (15.9)	15,548 (15.9)
25–29	113,300 (15.5)	98,131 (15.5)	15,169 (15.5)
30–34	102,670 (14.1)	88,818 (14.1)	13,852 (14.1)
35–39	99,206 (13.6)	85,959 (13.6)	13,247 (13.5)
40–44	85,412 (11.7)	73,966 (11.7)	11,446 (11.7)
45–49	81,158 (11.1)	70,426 (11.1)	10,732 (11.0)
Education, no. (%)			
Primary school or less	285,263 (39.1)	261,104 (41.3)	24,159 (24.7)
Secondary school unfinished	293,994 (40.3)	247,058 (39.1)	46,936 (47.9)
Secondary school finished or above	150,572 (20.6)	123,663 (19.6)	26,909 (27.5)
Household wealth index quintile, no. (%)			
Q1 (least wealthy)	134,810 (18.5)	117,732 (18.6)	17,078 (17.4)
Q2	145,106 (19.9)	125,974 (19.9)	19,132 (19.5)
Q3	150,502 (20.6)	130,348 (20.6)	20,154 (20.6)
Q4	148,048 (20.3)	127,521 (20.2)	20,527 (20.9)
Q5 (most wealthy)	151,363 (20.7)	130,250 (20.6)	21,113 (21.5)
Currently married, no. (%)	501,079 (68.7)	441,972 (70.0)	59,107 (60.3)
Urban area, no. (%)	215,231 (29.5)	184,532 (29.2)	30,699 (31.3)

These numbers were not weighted using sampling weights and represent all participants who had a non-missing value for the blood glucose measurement and fasting variable. Sample characteristics stratified by whether the blood glucose measurement or fasting status was missing are shown in Additional file 1: Table S1. The education group “Primary school or less” includes individuals without formal education, individuals who went to primary school but did not finish, and individuals who completed primary school

### Diabetes care cascade at the national level

The weighted prevalence of diabetes in the age group 15–49 years was 3.3% (95% CI, 3.2–3.4%), with a prevalence of 3.0% (95% CI, 2.9–3.1%) among women and 3.6% (95% CI, 3.3–3.8%) among men. Diabetes prevalence ranged from 0.6% (95% CI, 0.5–0.7%) among women aged 15 to 19 years to 9.6% (95% CI, 8.7–10.5%) among men aged 45–49 years (Additional file 1: Table S2).

**Fig. 1:**
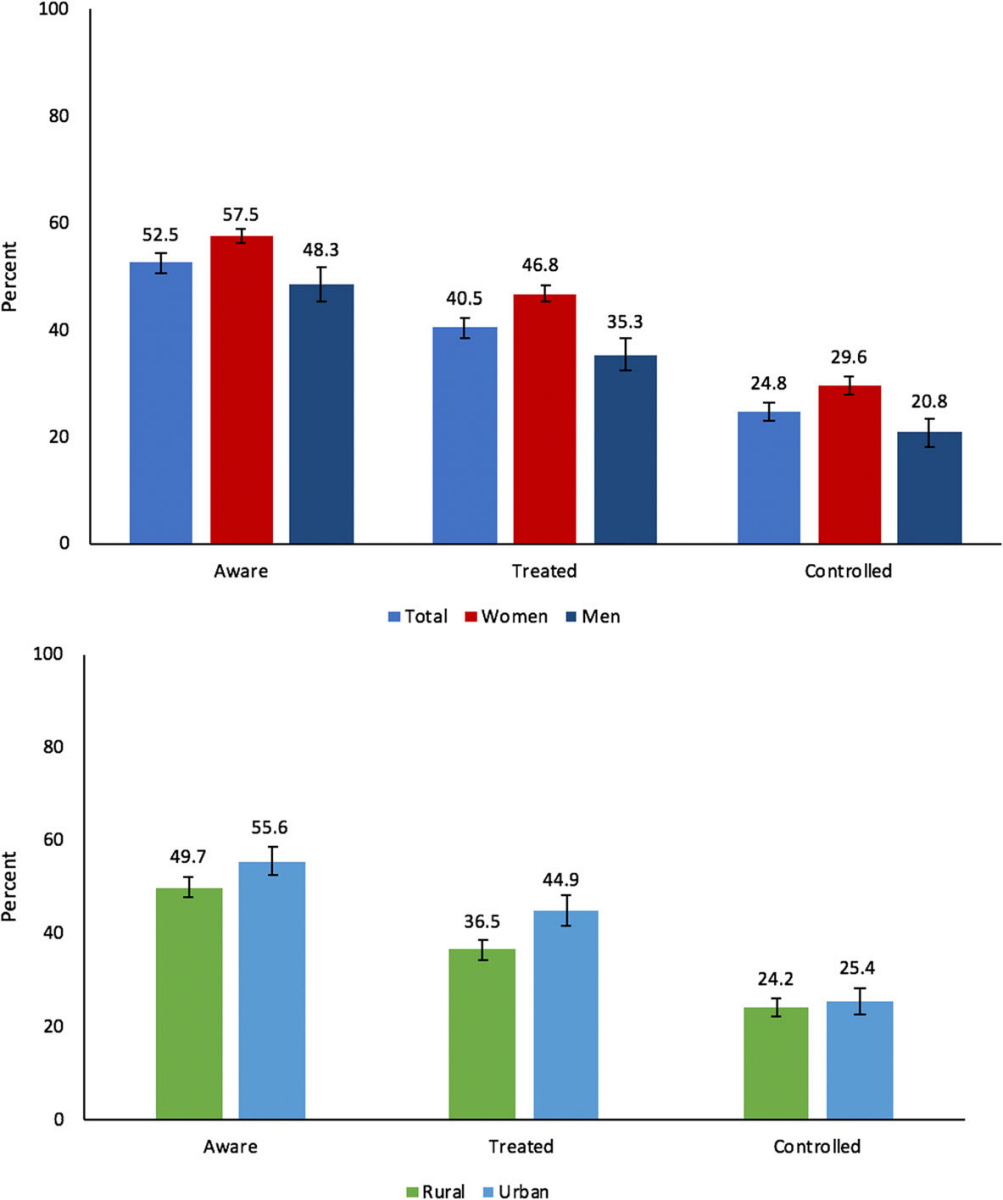
The cascade of care for diabetes in India. A flowchart of the cascade of care can be found in Additional file 1: Figure S5. Nineteen thousand four hundred fifty-three individuals with diabetes were included in this figure; 10,504 were “aware”, 8269 “treated”, and 5329 “controlled”

52.5% (95% CI, 50.6–54.4%) of those with diabetes were aware of their condition, 40.5% (95% CI, 38.6–42.3%) had sought treatment, and 24.8% (95% CI, 23.1–26.4%) were “controlled” (Fig. 1). Men and participants living in rural areas had worse outcomes for all diabetes care indicators (except for diabetes control for rural versus urban areas) than women and those living in urban areas. Care cascade indicators by 5-year age groups are shown in Additional file 1: Table S3.

### Diabetes care cascade by state

**Fig. 2:**
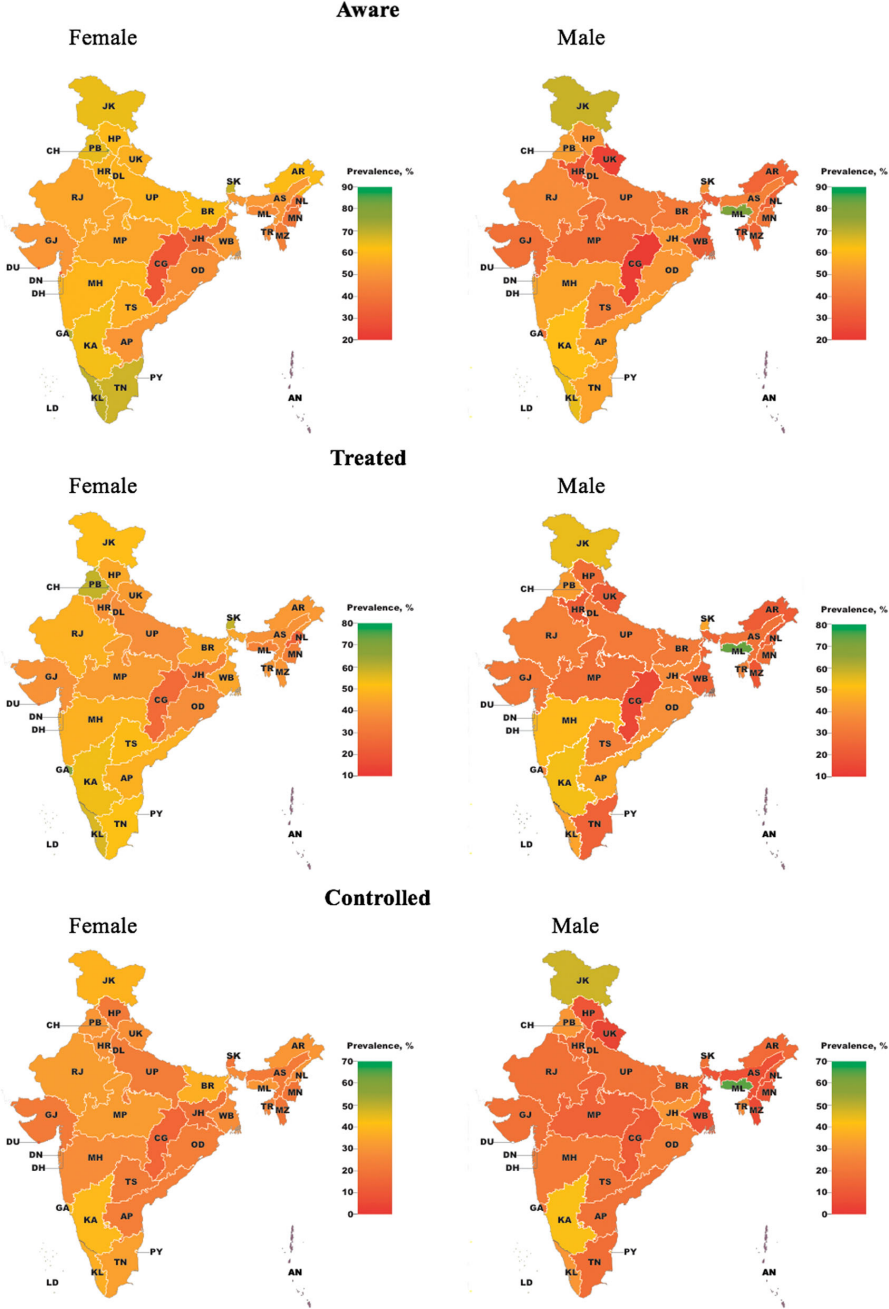
State-level variation in diabetes awareness, treatment, and control. Estimates with 95% confidence intervals are shown in Additional file 1: Table S10-S12. Diabetes prevalence estimates by sex and state are shown in Additional file 1: Table S13. Nineteen thousand four hundred fifty-three individuals with diabetes were included in this figure; 10,504 were “aware,” 8269 “treated,” and 5329 “controlled”

**Fig. 3:**
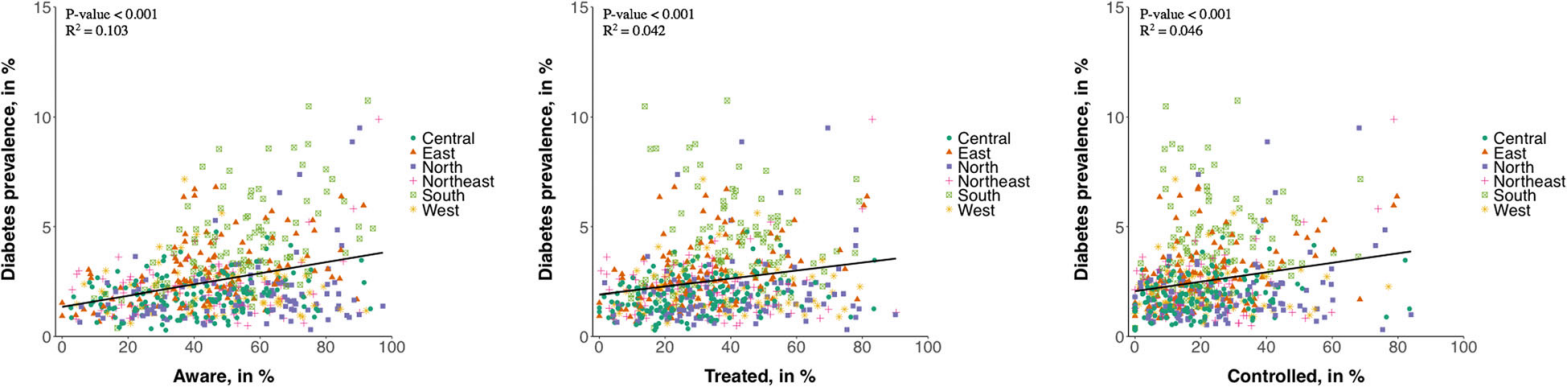
The association between district-level diabetes prevalence and care cascade indicators. All estimates were age-standardized to the Global Burden of Disease Project’s age structure for India for 2015 [23]. The same figure drawn separately for the age groups 15–29 years, 30–39 years, and 40–49 years can be found in Additional file 1: Figure S2. *P* values indicate the statistical significance of the slope of the regression line shown in black, which is an ordinary least squares regression of district-level diabetes prevalence onto district-level awareness/treatment among those with diabetes. *R*^2^ values are from the same regression. Nineteen thousand four hundred fifty-three individuals with diabetes were included in this figure; 10,504 were “aware”, 8269 “treated”, and 5329 “controlled”

Among states (excluding Union Territories), diabetes prevalence in the age group 15–49 years ranged from 1.6% (95% CI, 1.4–1.9%) in Rajasthan to 7.3% (95% CI, 5.4–9.7%) in Goa (Additional file 1: Table S4) and was generally highest in South India (Additional file 1: Figure S1). As shown in Fig. 2 and Additional file 1: Table S5-S7, the prevalence of being aware of one’s diabetes diagnosis ranged from 25.3% (95% CI, 19.4–32.3%) among those with diabetes in Chhattisgarh to 69.6% (95% CI, 52.6–82.6%) in Meghalaya. Among those with diabetes, having sought treatment varied from 19.7% (95% CI, 14.8–25.8%) in Chhattisgarh to 60.9% (95% CI, 45.1–74.8%) in Meghalaya, and the prevalence of controlled diabetes ranged from 13.0% (95% CI, 7.9–20.6%) in Nagaland to 53.7% (95% CI, 38.5–68.2%) in Meghalaya. There was a tendency for districts and states with a higher diabetes prevalence to have better care cascade indicators (Fig. 3 and Additional file 1: Figure S3).

The percentage of the total population in a state who had diabetes but was both unaware and untreated ranged from 0.8% (95% CI, 0.7–0.9%) in Rajasthan to 4.0% (95% CI, 3.0–5.0%) in Goa (Additional file 1: Table S8). The absolute number of adults with diabetes who was both unaware and untreated was highest in West Bengal (1,284,631; 95% CI, 1,119,388–1,442,204), Uttar Pradesh (1,225,532; 95% CI, 1,136,322–1,314,045), and Tamil Nadu (1,039,484; 95% CI, 888,052–1,199,337) (Additional file 1: Table S9).

### Diabetes care indicators by individuals’ socio-demographic characteristics

**Fig. 4:**
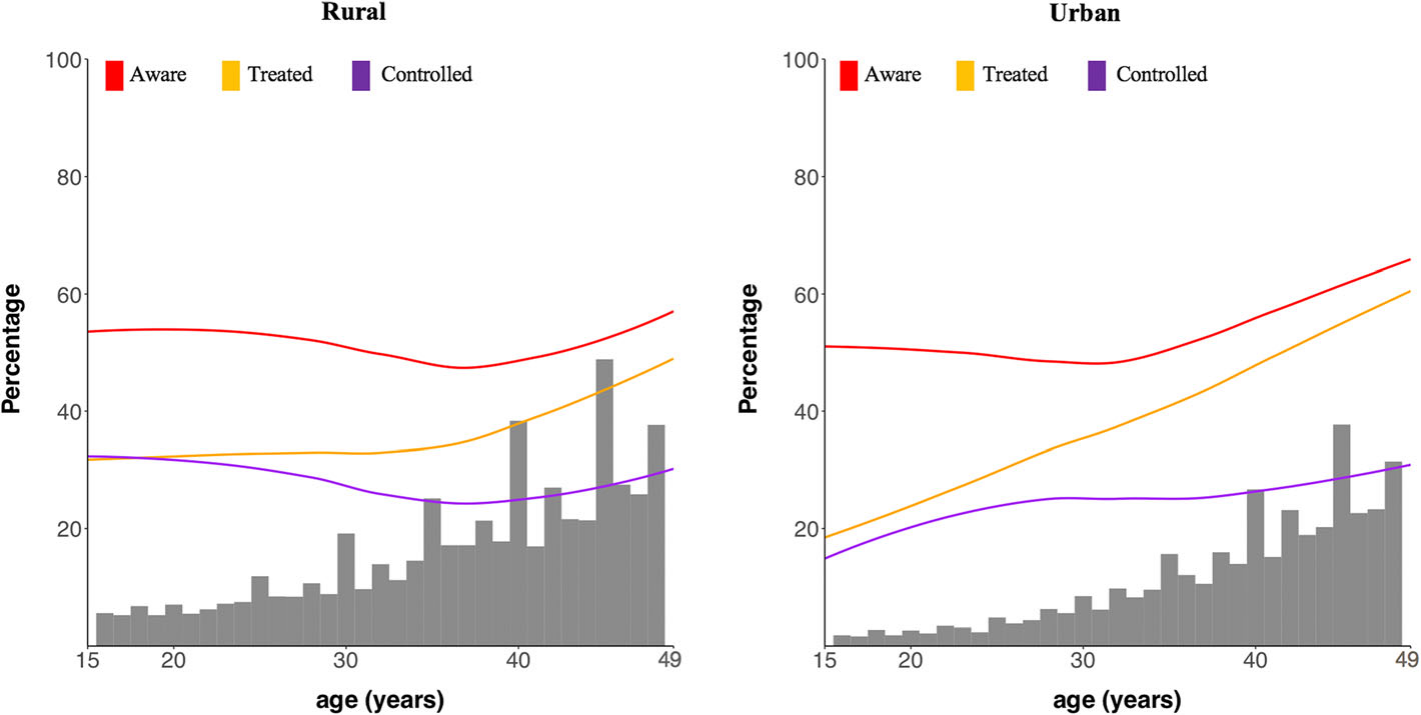
The predicted probability of reaching each cascade step by age as a continuous variable. Predicted probabilities were average adjusted predictions obtained from covariate-adjusted logistic regressions of diabetes care indicators on individuals’ socio-demographic characteristics (age, household wealth quintile, education, marital status, sex) and district-level fixed effects. We used restricted cubic splines with five knots for the continuous variable age. The knots were placed at the 5th, 27.5th, 50th, 72.5th, and 95th percentiles. Nineteen thousand four hundred fifty-three individuals with diabetes were included in this figure; 10,504 were “aware”, 8269 “treated”, and 5329 “controlled”

Covariate-adjusted logistic regressions of diabetes care indicators on individual’s socio-demographic characteristics (Table 2) show that being female, educational attainment, and a higher household wealth quintile were generally positively associated with better diabetes care indicators in both rural and urban areas. Being currently married was positively associated with each diabetes care cascade indicator in urban areas but negatively (except for the “controlled” step for which there was no association) in rural areas. Older age was more strongly associated with reaching each cascade step in urban than in rural areas, with the most positive association existing for the treatment step (Fig. 4). Additional file 1: Figure S4 shows that both the absolute and relative differences between household wealth index quintiles in the probabilities of being aware of one’s diabetes and having sought treatment increased with age group.

**Table 2:** Covariate-adjusted logistic regressions of diabetes care cascade indicators on socio-demographic characteristics

	Aware				Treated				Controlled			
	Rural		Urban		Rural		Urban		Rural		Urban	
	OR (95% CI)	*P*	OR (95% CI)	*P*	OR (95% CI)	*P*	OR (95% CI)	*P*	OR (95% CI)	*P*	OR (95% CI)	*P*
Education		Trend < 0.001		Trend < 0.001		Trend < 0.001		Trend < 0.001		Trend = 0.359		Trend < 0.001
Primary school or less	1 (reference)		1 (reference)		1 (reference)		1 (reference)		1 (reference)		1 (reference)	
Secondary school unfinished	1.18 (1.09–1.28)	< 0.001	1.13 (1.03–1.24)	0.007	1.35 (1.24–1.46)	< 0.001	1.35 (1.23–1.48)	< 0.001	1.01 (0.92–1.11)	0.785	0.95 (0.86–1.06)	0.355
Secondary school or above	1.23 (1.11–1.37)	< 0.001	1.59 (1.43–1.77)	< 0.001	1.26 (1.13–1.41)	< 0.001	1.63 (1.46–1.81)	< 0.001	1.06 (0.94–1.20)	0.359	1.59 (1.41–1.79)	< 0.001
Household wealth quintile		Trend = 0.001		Trend < 0.001		Trend < 0.001		Trend < 0.001		Trend < 0.001		Trend < 0.001
Q1 (poorest)	1 (reference)		1 (reference)		1 (reference)		1 (reference)		1 (reference)		1 (reference)	
Q2	0.80 (0.71–0.92)	0.001	1.17 (1.03–1.32)	0.015	0.87 (0.76–1.00)	0.050	1.27 (1.11–1.46)	< 0.001	1.00 (0.86–1.16)	0.998	0.92 (0.78–1.08)	0.287
Q3	0.89 (0.79–1.02)	0.093	1.24 (1.09–1.41)	0.001	1.06 (0.92–1.21)	0.431	1.85 (1.62–2.11)	< 0.001	1.09 (0.94–1.27)	0.242	1.43 (1.22–1.66)	< 0.001
Q4	1.02 (0.90–1.16)	0.741	1.01 (0.89–1.15)	0.870	1.40 (1.22–1.60)	< 0.001	1.50 (1.31–1.72)	< 0.001	1.14 (0.98–1.32)	0.093	1.16 (0.99–1.36)	0.062
Q5 (richest)	1.15 (1.00–1.32)	0.044	1.68 (1.45–1.93)	< 0.001	1.71 (1.48–1.98)	< 0.001	2.45 (2.12–2.84)	< 0.001	1.32 (1.13–1.54)	0.001	1.59 (1.35–1.88)	< 0.001
Currently married	0.82 (0.74–0.92)	< 0.001	1.13 (1.01–1.27)	0.027	0.86 (0.77–0.96)	0.008	1.45 (1.29–1.62)	< 0.001	0.98 (0.87–1.11)	0.730	1.62 (1.41–1.86)	< 0.001
Female	1.40 (1.31–1.50)	< 0.001	1.85 (1.72–1.99)	< 0.001	1.60 (1.49–1.72)	< 0.001	1.94 (1.80–2.09)	< 0.001	1.55 (1.44–1.68)	< 0.001	1.79 (1.65–1.94)	< 0.001

*Abbreviations*: *OR* odds ratio, *CI* confidence interval, *Q* quintile

These regressions contained all socio-demographic variables listed in the table (wealth quintile, education, marital status, and sex), age as a continuous variable with restricted cubic splines with five knots (the knots were placed at the 5th, 27.5th, 50th, 72.5th, and 95th percentiles), and a binary indicator for each district (district-level fixed effects) as explanatory variables. The regressions were weighted using sampling weights. Results for regressions run without sampling weights, not stratified by rural versus urban areas (but including rural/urban as a socio-demographic variable and interaction terms for education-rural/urban location and household wealth quintile-rural/urban location) and separately for women and men can be found in Additional file 1: Table S14-S18. Nineteen thousand four hundred fifty-three individuals with diabetes were included in the regressions for this table; 10,504 were “aware”, 8269 “treated”, and 5329 “controlled”. The *P* value for trend is for a linear trend

## Discussion

Using data from 729,829 individuals aged 15–49 years in India, we found that 3.3% (95% CI, 3.2–3.4%) had diabetes, of whom 52.5% (95% CI, 50.6–54.4%) were aware of their condition, 40.5% (95% CI, 38.6–42.3%) had sought treatment, and 24.8% (95% CI, 23.1–26.4%) had sought treatment and had a random plasma-equivalent blood glucose below the threshold for diabetes (“controlled”). Thus, across the care cascade from “aware” to “control,” a total of 75% of participants with diabetes were “lost” to care, 47% at the awareness stage, 12% at the treatment stage, and 16% due to failure to achieve control despite having sought treatment.

Hence, while in the Indian health system a substantial proportion of patients are lost to care at each step of the diabetes care cascade, there appears to be a particularly high need for improved detection of diabetes. Several large-scale efforts to improve diabetes screening in India have recently begun, such as the 5-year “UDAY” program in the states of Andhra Pradesh and Haryana [24]. Even though improved detection of diabetes cases seems essential, we would like to note that, thus far, no study in high-income countries has shown that screening alone improves diabetes-related outcomes [25]. In addition, a crucial question for the success of large-scale diabetes screening programs in India will be whether the health system is able to effectively cope with the resulting increase in demand for diabetes care.

We also identified important variation in the care cascade among population groups with indicators being worse for rural areas, men, those with less education, and—particularly among older age groups—those with lower household wealth. It is possible that some of the better health system performance for diabetes among women than men is explained by routine screening for gestational diabetes during antenatal care. The relatively low health system performance for diabetes among the rural poor is concerning from a health equity perspective given that these individuals (i) likely have the lowest access to high-quality care for the micro- and macrovascular complications of diabetes, (ii) are most likely to experience catastrophic healthcare expenditures from these complications [26, 27], and (iii) are most reliant on their physical health to earn their livelihood given that many of these individuals are subsistence farmers [28]. In addition to a focus on men, interventions to improve health system performance for diabetes are, therefore, particularly needed among rural populations with little education and household wealth.

The diabetes care cascade varied widely among states in India, with states with a lower prevalence of diabetes tending to have poorer diabetes care indicators, possibly because many of these—often less wealthy—states’ health systems largely focus on communicable diseases and maternal and child health [29], which still cause an important disease burden in India [30]. Nonetheless, given their population size, these states frequently have a large number of adults with diabetes. For example, Uttar Pradesh—a state with one of the lowest prevalence levels of diabetes in India—had one of the largest absolute numbers of adults who were unaware of their diabetes diagnosis (1,225,532; 95% CI, 1,136,322–1,314,045). While diabetes may not be a public health priority for these states, improvements in diabetes care are still essential in these states to increase health system performance for diabetes in India at the national level. As a percentage of the total adult population aged 15–49 years, the states and Union Territories with the highest proportion of adults who had diabetes but had never sought diabetes treatment were Goa (4.2%; 95% CI, 3.2–5.2%), Tamil Nadu (3.8%; 95% CI, 3.4–4.1%), and Andaman and Nicobar Islands (3.7%; 95% CI, 2.7–4.8%), demonstrating a particularly high need to intensify diabetes detection and care programs in these states.

To our knowledge, only three other studies—from Malawi [13], South Africa [14], and the USA [15]—have assessed the diabetes care cascade in a nationwide sample. While these studies differed in their definition of individual cascade steps, the proportion of adults with diabetes who were aware of their diagnosis was substantially lower in India (52.5% [95% CI, 50.6–54.4%]) than in the USA (72.2%), but comparable to that found in South Africa (45.4%) and Malawi (41.2%). Comparing our results to the ICMR-INDIAB study, which has published relevant data on 14 of 29 states and one of seven Union Territories in India, we find that our estimate of being aware of one’s diabetes diagnosis was very similar (52.7% in ICMR-INDIAB versus 52.5% [95% CI, 50.6–54.4%] in this analysis) [18, 31, 32]. As in this study, ICMR-INDIAB found awareness of one’s diabetes diagnosis to be lower in rural than in urban areas (with a ratio of self-reported diabetes to newly diagnosed diabetes in rural areas of 1:1.5 compared to 1:0.7 in urban areas) [18]. Glycemic control estimates obtained from a subsample of 14,200 adults with diabetes in ICMR-INDIAB were somewhat higher than those in this study (31% in ICMR-INDIAB versus 24.8% [95% CI, 23.1–26.4%] in our analysis) [17], likely at least partially due to the fact that ICMR-INDIAB was able to assess glycemic control using a HbA1c measurement while we had to rely on random blood glucose.

This analysis has highlighted the great potential for improving diabetes control among adults aged 15–49 years with diabetes in India. An additional—and possibly more cost-effective—strategy to reducing the impact of diabetes on population health in India is primary prevention efforts. The reasons for the rapid rise of diabetes in the Indian population are not entirely understood but likely include (i) population aging [1], (ii) change of lifestyles from manual labor to sedentary work environments for an increasing proportion of Indians [33, 34], (iii) lack of green space for physical exercise in many urban areas [35], and (iv) changing dietary patterns [33, 36]. Further factors that have been linked to diabetes and appear particularly pertinent to India include air pollution [37, 38], low birth weight (which is comparatively prevalent in India) [39–42], and a genetic predisposition towards developing diabetes among South Asians [43–46]. While reforms and interventions to improve health services for diabetes in India will be important, we believe that a successful response to India’s diabetes epidemic should also include efforts to address some of these underlying reasons for the rise of diabetes in the population. Such efforts may include information campaigns, changes in the physical environment to improve opportunities for physical exercise, innovations in the transport system and regulatory reform to curb air pollution, and interventions to improve the availability and affordability of healthy foods.

This study has several limitations. First, for the vast majority of participants (98.9%), diabetes was defined based on a random blood glucose measurement. According to the International Diabetes Federation, a random blood glucose measurement ≥ 200 mg/dL would have to be measured in a symptomatic patient in order to be considered as a diabetes-defining criterion [22]. We, however, had no data on diabetes symptoms. Given that the random blood glucose measurement likely misclassified a substantial number of adults without diabetes as having diabetes (and that we would not expect those without diabetes to have previously been told to have diabetes or to be on treatment), this imperfect measurement of diabetes probably biased the care cascade indicators downwards. Second, glycemic control should ideally be assessed with an HbA1c measurement rather than a random blood glucose measurement. Apart from adding noise to our “controlled” estimates and thus resulting in less precise regression estimates for that cascade step, it is unclear whether and how this limitation biased our results. Third, treatment of diabetes was ascertained through the question “Have you sought treatment for this issue [diabetes]?” which did not allow us to assess what (if any) treatment was prescribed (e.g., oral medications, insulin, and/or advice to change lifestyle behaviors) and whether the participant was still taking the treatment at the time of the survey. This limitation highlights the need to improve questionnaire design for large-scale government-led studies in India. To aid comparison over time and across countries, it would also be useful for future assessments of cardiometabolic disease care to use a standardized set of questions. Fourth, eligibility for this survey was restricted to those aged 15–49 years, which implies that our estimates of diabetes prevalence and care cascade indicators are not representative for the entire adult population. It is likely that this age restriction, along with the use of a random instead of a fasting plasma glucose, largely explains the lower prevalence of diabetes in this study compared to that in a recently published older sample of the Indian population [4], as well as the IMCR-INDIAB study [18]. Fifth, we were not able to differentiate between type 1 and type 2 diabetes in this study. However, it is very likely that the vast majority of those with diabetes in this analysis had type 2 diabetes. According to the International Diabetes Federation, an estimated 72,000 children aged 0 to 14 years lived with type 1 diabetes in India in 2015, which corresponds to just 0.02% of the country’s population in this age range [2, 47]. Lastly, men were underrepresented in this analysis, constituting only 13.4% of the total sample. However, the sample still contains a large absolute number of men (98,004), thus allowing for reasonably precise estimates of diabetes prevalence and care for men. We corrected for the lower probability of selecting men in this survey through weighting. In addition, we show all results (either in the main manuscript or in the appendix) disaggregated by sex.

## Conclusions

This large population-based analysis of adults aged 15–49 years found substantial losses of patients at each step of the diabetes care cascade, with the highest proportion (47%) being lost to care at the awareness stage. Improvements in health system performance for diabetes in India are particularly needed for rural areas, those with little wealth and education, and men. While adults with undiagnosed diabetes make up the largest proportion of the state’s population in Goa and Andhra Pradesh, efforts at improving diabetes care should not neglect large states with a low diabetes prevalence (e.g., Uttar Pradesh), which host some of the highest absolute numbers of adults who are unaware of their diabetes and untreated. India’s success in coping with the consequences of its diabetes epidemic will in an important part depend on its ability to improve health system performance for diabetes. “Sealing the leaks” of the diabetes care cascade will thus be a crucial determinant of the country’s ability to reach the SDG target of reducing premature mortality from NCDs by one third by 2030 [7]. Given the country’s size and projected population growth, India’s performance in achieving this target will ultimately also have a decisive influence on the world’s ability to reach this SDG.

## Additional file

Additional file 1: Supplementary tables and figures referred to in the manuscript. (DOCX 3029 kb)

## Abbreviations

CVD: Cardiovascular disease
HIV: Human immunodeficiency virus
NCD: Non-communicable disease
NFHS-4: Fourth National Family Health Survey
PSU: Primary sampling unit
RBG: Random blood glucose
SDG: Sustainable Development Goal
UN: United Nations
WHO: World Health Organization
