# Supplementary material for: Variation in health system performance for managing diabetes among states in India: a cross-sectional study of individuals aged 15 to 49 years
Source: BMC Med. 2019 May 13;17:92. doi: 10.1186/s12916-019-1325-6 (PMC6515628; doi:10.1186/s12916-019-1325-6)
Supplement: Supplementary file 1 — Supplementary tables and figures referred to in the manuscript. (DOCX 3029 kb) [file 12916_2019_1325_MOESM1_ESM.docx]

**Supplementary Appendix**

[Methods S1. Methodology of the NFHS-4 3](#_Toc4179466)

[Methods S2. Computation of unmet need for care variables 5](#_Toc4179467)

[Methods S3. Computation of the household wealth index 6](#_Toc4179468)

[Table S1. Sample characteristics stratified by whether the blood glucose measurement was missing^a^ 8](#_Toc4179469)

[Table S2. Diabetes prevalence (in %) by five-year age group and sex 9](#_Toc4179470)

[Table S3. Care cascade indicators (in %) by five-year age group 10](#_Toc4179471)

[Table S4. State-level diabetes prevalence estimates 11](#_Toc4179472)

[Table S5. State-level estimates for being aware of one’s diabetes diagnosis 12](#_Toc4179473)

[Table S6. State-level estimates for having sought treatment for diabetes 13](#_Toc4179474)

[Table S7. State-level estimates for having achieved diabetes control 14](#_Toc4179475)

[Table S8. Estimated percentage of total population per state with unmet need for diabetes care^a^ 15](#_Toc4179476)

[Table S9. Total number of individuals with unmet need for care by state^a^ 17](#_Toc4179477)

[Table S10. State-level prevalence estimates of ‘aware’, by sex 20](#_Toc4179478)

[Table S11. State-level prevalence estimates of ‘treated’, by sex 22](#_Toc4179479)

[Table S12. State-level prevalence estimates of ‘controlled’, by sex 24](#_Toc4179480)

[Table S13. State-level diabetes prevalence estimates, by sex 26](#_Toc4179481)

[Table S14. Multivariable logistic regressions of diabetes care indicators on socio-demographic characteristics without sampling weights^a^ 28](#_Toc4179482)

[Table S15. Multivariable (weighted) logistic regressions of diabetes care indicators on socio-demographic characteristics, with an interaction term between household wealth quintile and rural/urban location^a^ 29](#_Toc4179483)

[Table S16. Multivariable (weighted) logistic regressions of diabetes care indicators on socio-demographic characteristics, with an interaction term between education and rural/urban location^a^ 30](#_Toc4179484)

[Table S17. Multivariable logistic regressions of diabetes care indicators on socio-demographic characteristics, among women only^a^ 31](#_Toc4179485)

[Table S18. Multivariable logistic regressions of diabetes care indicators on socio-demographic characteristics, among men only^a^ 32](#_Toc4179486)

[Figure S1. Map of diabetes prevalence by state, age group 15 - 49 years 33](#_Toc4179487)

[Figure S2. The association between district-level diabetes prevalence and care cascade indicators, separately for age group 15 - 29 years, 30 - 39 years, and 40 - 49 years ^a,b,c^ 34](#_Toc4179488)

[Figure S3. The association between state-level diabetes prevalence and care cascade indicators^a,b,c,d^ 35](#_Toc4179489)

[Figure S4. Predicted probabilities of awareness, treatment, and control by age group, rural or urban location, and household wealth quintile^a^ 36](#_Toc4179490)

[Figure S5. Flowchart of the proportion of patients lost in the diabetes care cascade 37](#_Toc4179491)

[References 38](#_Toc4179492)

# Methods S1. Methodology of the NFHS-4

The NFHS-4 was initiated, as all NFHS surveys before, by the Ministry of Health and Family Welfare (MoHFW) and carried out by the International Institute for Population Sciences (IIPS) in Mumbai as the nodal agency. Funding came from multiple international governmental and non-governmental organizations.

The sampling process differed in rural and urban areas, with villages as primary sampling units (PSUs) in rural areas (selected with probability proportional to size) and census enumeration blocks (CEBs) as PSUs in urban areas (selected through random sampling). Small PSUs with fewer than 40 households were added to the nearest PSU, whereas large PSUs with more than 300 households were divided into segments of 100-150 households. In each of these clusters (PSUs or PSU segments) 22 households were selected for the final survey using systematic random sampling. Households were revisited up to three times in case that i) an eligible household member was not available; ii) no one was present in the household at the time of the visit.

Four Computer Assisted Personal Interviewing (CAPI) questionnaires - household, man’s, woman’s and biomarker - were used with women aged 15 - 49 and men aged 15 - 54 years. For this analysis we excluded men aged 50 - 54 years to have equal age ranges among men and women. In the household questionnaire, general information about the socioeconomic characteristics of the household (all usual members and visitors that had stayed the previous night) was collected. The woman’s questionnaire was distributed in two versions with the first version including all information used in this analysis on district, state and national levels, whereas the second version was only distributed among a subsample providing information only on the state and national level and included four additional topics not used for this analysis. The man’s questionnaire was only distributed in one version and contained all the information used in this analysis on district, state and national levels.

The woman’s and man’s questionnaires both contained the key questions used in this survey, “Do you currently have diabetes?” and “Have you sought treatment for this problem?”. The biomarker questionnaire contained the measurement of blood glucose and also the questions “When was the last time you had something to eat?” and “When was the last time you had something to drink other than plain water?” that were essential to apply the glucose reading interpretation according to the recommendations of the International Diabetes Federation and the World Health Organization (WHO).[[1](#_ENREF_1)]

More detailed information on i) the sampling procedure; ii) the technical training of the staff; iii) pre-survey pilot studies; iv) all questionnaire items and biomarker measurements; v) the execution of the survey can be found in the formal report of the NFHS-4, the published questionnaires and the multiple manuals that were distributed to the staff, which are all available online.[[2-9](#_ENREF_2)]

# Methods S2. Computation of unmet need for care variables

For Table S7 and Table S11, we performed several steps to convert the cascade of care analysis into unmet need for care population percentages for each state (Table S7) and absolute number estimates of unmet need for care for each state (Table S11). First, we calculated the reciprocal value of each diabetes care indicator to convert the cascade of care estimates into unmet need for care estimates (Unaware, Untreated, Uncontrolled). Since there were no missing values for any of the diabetes care indicators in the diabetic population, we did not adjust for missing values. Second, we multiplied the new unmet need for care values with the diabetes prevalence per state to calculate state percentages of each unmet need for care estimate (Table S7). Third, we multiplied each percentage of step two with the population of each state of age 15-49 years (single year numbers of the national census 2011 were used to calculate state population for the analyzed age group) to estimate absolute numbers of the people in a state who were unaware, untreated, and uncontrolled (Table S11).

# Methods S3. Computation of the household wealth index

We used the household wealth index quintiles given in the DHS data. The DHS program has published detailed information on the computation of the household wealth index elsewhere.[[7](#_ENREF_7), [10](#_ENREF_10)] The data used to construct the household wealth index included measures of a household’s key characteristics and ownership of durable goods. The following key housing characteristics were used:

- Main roof material
- Main wall material
- Main material of floor
- Type of cooking fuel
- Type of toilet facility
- Source of drinking water

In addition, ownership of the following durable goods (‘assets’) was used:

| - Mattress |
| --- |
| - Pressure cooker |
| - Chair |
| - Cot or bed |
| - Table |
| - Electric fan |
| - Radio or transistor |
| - Black and white television |
| - Colour television |
| - Sewing machine |
| - Mobile telephone |
| - Telephone (non-mobile) |
| - Internet |
| - Computer |
| - Refrigerator |
| - Air conditioner/cooler |
| - Washing machine |
| - Watch or clock |
| - Bicycle |
| - Motorcycle or Scooter |
| - Animal-drawn cart |
| - Car |
| - Water pump |
| - Thresher |
| - Tractor |

First, a binary indicator for each household characteristic and asset was created. Second, separately for rural and urban areas, the data was summarized using the first component (unrotated) of a principal component analysis (PCA). The resulting asset ‘score’ has a mean of zero and standard deviation of one with higher values indicating more wealth. The advantage of this approach is that the weights attributed to each housing characteristic and asset are data-driven instead of assigned arbitrarily. Third, again separately for rural and urban areas, the continuous household wealth index variable was divided into quintiles for easier interpretability.

# Table S1. Sample characteristics stratified by whether the blood glucose measurement was missing^a^

| **Characteristic** | **Missing** | **Not missing** |
| --- | --- | --- |
| No. | 19,290 | 729,829 |
| Male, no. (%) | 3,664 (19.0) | 98,004 (13.4) |
| Age group, no. in years (%) |  |  |
| 15-19 | 3,985 (20.7) | 131,984 (18.1) |
| 20-24 | 3,232 (16.8) | 116,099 (15.9) |
| 25-29 | 3,031 (15.7) | 113,300 (15.5) |
| 30-34 | 2,533 (13.1) | 102,670 (14.1) |
| 35-39 | 2,363 (12.2) | 99,206 (13.6) |
| 40-44 | 2,107 (10.9) | 85,412 (11.7) |
| 45-49 | 2,039 (10.6) | 81,158 (11.1) |
| Education, no. (%) |  |  |
| Primary school or less | 6,174 (32.0) | 285,263 (39.1) |
| Secondary school unfinished | 7,704 (39.9) | 293,994 (40.3) |
| Secondary school finished or above | 5,412 (28.1) | 150,572 (20.6) |
| Household wealth index quintile, no. (%) |  |  |
| Q1 (Poorest) | 3,170 (16.4) | 134,810 (18.5) |
| Q2 | 3,412 (17.7) | 145,106 (19.9) |
| Q3 | 3,801 (19.7) | 150,502 (20.6) |
| Q4 | 3,999 (20.7) | 148,048 (20.3) |
| Q5 (Richest) | 4,908 (25.4) | 151,363 (20.7) |
| Currently married, no. (%) | 11,678 (60.5) | 501,079 (68.7) |
| Urban area, no. (%) | 8,584 (44.5) | 215,231 (29.5) |

Abbreviations: no.=number; %=Percentage.

^a^ These numbers were not weighted using sampling weights.

# Table S2. Diabetes prevalence (in %) by five-year age group and sex

| Age group (years) | Total (95% CI) | Women (95% CI) | Men (95% CI) |
| --- | --- | --- | --- |
| 15-19 | 0.6 (0.5-0.7) | 0.6 (0.5-0.7) | 0.6 (0.5-0.8) |
| 20-24 | 0.9 (0.8-0.10) | 0.9 (0.8-0.10) | 0.9 (0.7-0.12) |
| 25-29 | 1.5 (1.4-1.7) | 1.5 (1.3-1.6) | 1.6 (1.3-1.9) |
| 30-34 | 2.6 (2.4-2.8) | 2.3 (2.2-2.5) | 2.9 (2.5-3.3) |
| 35-39 | 4.2 (3.9-4.5) | 3.7 (3.5-3.9) | 4.7 (4.2-5.3) |
| 40-44 | 7.0 (6.5-7.5) | 6.0 (5.7-6.3) | 7.9 (7.0-8.8) |
| 45-49 | 9.2 (8.7-9.7) | 8.9 (8.5-9.3) | 9.6 (8.7-10.5) |

# Table S3. Care cascade indicators (in %) by five-year age group

*Awareness:*

| Age group | Aware, in % | Lower 95% CI | Upper 95% CI |
| --- | --- | --- | --- |
| 15-19 | 54.3 | 46.0 | 62.3 |
| 20-24 | 51.5 | 44.4 | 58.5 |
| 25-29 | 47.8 | 42.5 | 53.2 |
| 30-34 | 47.7 | 43.6 | 51.8 |
| 35-39 | 49.1 | 45.4 | 52.8 |
| 40-44 | 51.9 | 48.2 | 55.6 |
| 45-49 | 57.3 | 54.4 | 60.2 |

*Treatment:*

| Age group | Treated, in % | Lower 95% CI | Upper 95% CI |
| --- | --- | --- | --- |
| 15-19 | 27.0 | 20.7 | 34.4 |
| 20-24 | 25.5 | 20.1 | 31.8 |
| 25-29 | 28.7 | 24.3 | 33.5 |
| 30-34 | 33.3 | 29.7 | 37.0 |
| 35-39 | 35.7 | 32.5 | 39.0 |
| 40-44 | 43.1 | 39.4 | 46.8 |
| 45-49 | 49.4 | 46.5 | 52.3 |

*Control:*

| Age group | Controlled, in % | Lower 95% CI | Upper 95% CI |
| --- | --- | --- | --- |
| 15-19 | 25.1 | 19.0 | 32.3 |
| 20-24 | 23.3 | 18.0 | 29.5 |
| 25-29 | 25.4 | 21.2 | 30.1 |
| 30-34 | 23.5 | 20.4 | 26.9 |
| 35-39 | 20.4 | 17.9 | 23.1 |
| 40-44 | 24.9 | 21.8 | 28.3 |
| 45-49 | 27.4 | 24.9 | 30.0 |

# Table S4. State-level diabetes prevalence estimates

| State | Estimate | Lower 95% CI | Upper 95% CI |
| --- | --- | --- | --- |
| Andaman and Nicobar Islands | 7.7 | 6.0 | 9.9 |
| Andhra Pradesh | 5.7 | 4.9 | 6.6 |
| Arunachal Pradesh | 2.3 | 1.8 | 2.9 |
| Assam | 2.3 | 2.0 | 2.7 |
| Bihar | 2.8 | 2.5 | 3.1 |
| Chandigarh | 3.6 | 1.9 | 6.7 |
| Chhattisgarh | 2.6 | 2.2 | 3.1 |
| Dadra and Nagar Haveli | 3.4 | 1.9 | 5.9 |
| Daman and Diu | 2.6 | 1.2 | 5.5 |
| Delhi | 4.3 | 3.2 | 5.7 |
| Goa | 7.3 | 5.4 | 9.7 |
| Gujarat | 2.8 | 2.4 | 3.2 |
| Haryana | 1.7 | 1.4 | 2.1 |
| Himachal Pradesh | 2.5 | 2.1 | 3.1 |
| Jammu and Kashmir | 3.8 | 3.2 | 4.5 |
| Jharkhand | 2.8 | 2.4 | 3.2 |
| Karnataka | 4.1 | 3.3 | 5.1 |
| Kerala | 6.6 | 5.8 | 7.5 |
| Lakshadweep | 5.4 | 3.9 | 7.5 |
| Madhya Pradesh | 2.2 | 2.0 | 2.4 |
| Maharashtra | 2.3 | 2.0 | 2.7 |
| Manipur | 2.7 | 2.2 | 3.2 |
| Meghalaya | 4.0 | 2.6 | 6.2 |
| Mizoram | 2.9 | 2.3 | 3.6 |
| Nagaland | 2.6 | 2.1 | 3.2 |
| Odisha | 4.3 | 3.9 | 4.8 |
| Puducherry | 4.8 | 3.4 | 6.7 |
| Punjab | 2.6 | 2.3 | 3.0 |
| Rajasthan | 1.6 | 1.4 | 1.9 |
| Sikkim | 2.5 | 1.9 | 3.2 |
| Tamil Nadu | 6.0 | 5.3 | 6.8 |
| Telangana | 4.1 | 3.4 | 4.9 |
| Tripura | 4.0 | 3.1 | 5.1 |
| Uttar Pradesh | 2.2 | 2.0 | 2.4 |
| Uttarakhand | 2.7 | 2.2 | 3.2 |
| West Bengal | 4.0 | 3.4 | 4.6 |

# Table S5. State-level estimates for being aware of one’s diabetes diagnosis

| State | Estimate | Lower 95% CI | Upper 95% CI |
| --- | --- | --- | --- |
| Andaman and Nicobar Islands | 56.5 | 42.1 | 69.9 |
| Andhra Pradesh | 52.7 | 45.2 | 60.0 |
| Arunachal Pradesh | 46.4 | 35.6 | 57.6 |
| Assam | 47.7 | 41.4 | 54.1 |
| Bihar | 52.6 | 46.4 | 58.6 |
| Chandigarh | 59.5 | 33.3 | 81.2 |
| Chhattisgarh | 25.3 | 19.4 | 32.3 |
| Dadra and Nagar Haveli | 31.4 | 8.4 | 69.5 |
| Daman and Diu | 5.5 | 2.1 | 13.8 |
| Delhi | 47.5 | 33.9 | 61.5 |
| Goa | 45.0 | 31.8 | 58.9 |
| Gujarat | 41.8 | 34.2 | 49.9 |
| Haryana | 45.8 | 37.3 | 54.6 |
| Himachal Pradesh | 53.6 | 43.9 | 63.0 |
| Jammu and Kashmir | 67.8 | 58.7 | 75.7 |
| Jharkhand | 47.8 | 40.4 | 55.3 |
| Karnataka | 62.7 | 52.8 | 71.7 |
| Kerala | 66.8 | 60.1 | 72.8 |
| Lakshadweep | 55.3 | 33.1 | 75.5 |
| Madhya Pradesh | 45.8 | 41.1 | 50.5 |
| Maharashtra | 56.6 | 48.3 | 64.6 |
| Manipur | 40.4 | 32.4 | 49.0 |
| Meghalaya | 69.6 | 52.6 | 82.6 |
| Mizoram | 40.5 | 28.4 | 54.0 |
| Nagaland | 38.1 | 29.0 | 48.2 |
| Odisha | 50.5 | 45.6 | 55.4 |
| Puducherry | 57.0 | 42.8 | 70.2 |
| Punjab | 58.8 | 51.0 | 66.2 |
| Rajasthan | 50.4 | 43.3 | 57.6 |
| Sikkim | 58.8 | 44.4 | 71.8 |
| Tamil Nadu | 61.0 | 55.0 | 66.7 |
| Telangana | 51.8 | 41.2 | 62.2 |
| Tripura | 40.2 | 29.6 | 51.8 |
| Uttar Pradesh | 47.4 | 43.6 | 51.3 |
| Uttarakhand | 41.7 | 33.6 | 50.2 |
| West Bengal | 42.0 | 34.9 | 49.5 |

# Table S6. State-level estimates for having sought treatment for diabetes

| State | Estimate | Lower 95% CI | Upper 95% CI |
| --- | --- | --- | --- |
| Andaman and Nicobar Islands | 51.3 | 38.1 | 64.4 |
| Andhra Pradesh | 46.2 | 38.9 | 53.7 |
| Arunachal Pradesh | 30.8 | 22.5 | 40.6 |
| Assam | 33.7 | 27.8 | 40.1 |
| Bihar | 39.1 | 33.1 | 45.5 |
| Chandigarh | 54.6 | 27.3 | 79.3 |
| Chhattisgarh | 19.7 | 14.8 | 25.8 |
| Dadra and Nagar Haveli | 21.1 | 4.2 | 62.2 |
| Daman and Diu | 5.3 | 2.0 | 13.5 |
| Delhi | 43.2 | 30.1 | 57.4 |
| Goa | 42.0 | 29.4 | 55.8 |
| Gujarat | 34.5 | 27.4 | 42.3 |
| Haryana | 31.8 | 25.0 | 39.4 |
| Himachal Pradesh | 35.8 | 27.4 | 45.1 |
| Jammu and Kashmir | 54.4 | 46.1 | 62.5 |
| Jharkhand | 36.2 | 28.7 | 44.5 |
| Karnataka | 54.0 | 42.9 | 64.7 |
| Kerala | 50.9 | 44.9 | 56.9 |
| Lakshadweep | 44.3 | 24.4 | 66.2 |
| Madhya Pradesh | 35.4 | 31.4 | 39.5 |
| Maharashtra | 47.9 | 40.1 | 55.9 |
| Manipur | 31.4 | 23.7 | 40.3 |
| Meghalaya | 60.9 | 45.1 | 74.8 |
| Mizoram | 31.6 | 21.7 | 43.4 |
| Nagaland | 26.0 | 18.2 | 35.6 |
| Odisha | 37.9 | 33.3 | 42.8 |
| Puducherry | 49.0 | 36.5 | 61.5 |
| Punjab | 51.1 | 42.9 | 59.2 |
| Rajasthan | 39.5 | 33.3 | 46.2 |
| Sikkim | 52.1 | 38.9 | 65.0 |
| Tamil Nadu | 37.4 | 32.1 | 43.1 |
| Telangana | 42.9 | 33.4 | 53.0 |
| Tripura | 37.6 | 27.2 | 49.2 |
| Uttar Pradesh | 35.4 | 31.8 | 39.1 |
| Uttarakhand | 33.7 | 25.8 | 42.7 |
| West Bengal | 34.7 | 28.0 | 42.0 |

# Table S7. State-level estimates for having achieved diabetes control

| State | Estimate | Lower 95% CI | Upper 95% CI |
| --- | --- | --- | --- |
| Andaman and Nicobar Islands | 27.7 | 19.5 | 37.8 |
| Andhra Pradesh | 20.9 | 15.9 | 27.0 |
| Arunachal Pradesh | 23.3 | 16.2 | 32.3 |
| Assam | 20.5 | 15.8 | 26.1 |
| Bihar | 29.9 | 24.0 | 36.6 |
| Chandigarh | 27.0 | 7.7 | 62.3 |
| Chhattisgarh | 13.6 | 9.4 | 19.2 |
| Dadra and Nagar Haveli | 18.2 | 2.9 | 62.4 |
| Daman and Diu | 2.1 | 0.6 | 7.5 |
| Delhi | 17.5 | 10.3 | 28.2 |
| Goa | 21.7 | 13.2 | 33.6 |
| Gujarat | 19.0 | 13.6 | 26.0 |
| Haryana | 24.1 | 18.5 | 30.7 |
| Himachal Pradesh | 14.9 | 10.4 | 20.8 |
| Jammu and Kashmir | 45.1 | 36.7 | 53.7 |
| Jharkhand | 27.7 | 20.3 | 36.5 |
| Karnataka | 41.0 | 29.0 | 54.1 |
| Kerala | 34.0 | 29.3 | 39.1 |
| Lakshadweep | 19.4 | 8.9 | 37.3 |
| Madhya Pradesh | 22.8 | 19.6 | 26.4 |
| Maharashtra | 25.4 | 19.3 | 32.7 |
| Manipur | 14.4 | 9.7 | 20.9 |
| Meghalaya | 53.7 | 38.5 | 68.2 |
| Mizoram | 13.3 | 8.2 | 20.8 |
| Nagaland | 13.0 | 7.9 | 20.6 |
| Odisha | 24.1 | 19.8 | 29.0 |
| Puducherry | 33.0 | 22.7 | 45.4 |
| Punjab | 30.5 | 23.6 | 38.4 |
| Rajasthan | 23.9 | 18.9 | 29.8 |
| Sikkim | 20.1 | 11.5 | 32.6 |
| Tamil Nadu | 24.2 | 20.0 | 29.0 |
| Telangana | 21.1 | 14.5 | 29.7 |
| Tripura | 26.6 | 17.3 | 38.6 |
| Uttar Pradesh | 21.4 | 18.4 | 24.8 |
| Uttarakhand | 17.3 | 13.1 | 22.5 |
| West Bengal | 17.1 | 13.2 | 21.8 |

# Table S8. Estimated percentage of total population per state with unmet need for diabetes care^a^

|  | **Unaware** | | | **Untreated** | | | **Uncontrolled** | | | |
| --- | --- | --- | --- | --- | --- | --- | --- | --- | --- | --- |
|  | **Estimate** | **Lower 95% CI** | **Upper 95% CI** | **Estimate** | **Lower 95% CI** | **Upper 95% CI** | **Estimate** | **Lower 95% CI** | **Upper 95% CI** |  |
| Andaman and Nicobar Islands | 3.3 | 2.3 | 4.5 | 3.7 | 2.7 | 4.8 | 5.6 | 4.8 | 6.2 |  |
| Andhra Pradesh | 2.7 | 2.3 | 3.1 | 3.1 | 2.6 | 3.5 | 4.5 | 4.2 | 4.8 |  |
| Arunachal Pradesh | 1.2 | 1.0 | 1.5 | 1.6 | 1.4 | 1.8 | 1.8 | 1.6 | 1.9 |  |
| Assam | 1.2 | 1.1 | 1.3 | 1.5 | 1.4 | 1.7 | 1.8 | 1.7 | 1.9 |  |
| Bihar | 1.3 | 1.2 | 1.5 | 1.7 | 1.5 | 1.9 | 2.0 | 1.8 | 2.1 |  |
| Chandigarh | 1.5 | 0.7 | 2.4 | 1.6 | 0.7 | 2.6 | 2.6 | 1.4 | 3.3 |  |
| Chhattisgarh | 1.9 | 1.8 | 2.1 | 2.1 | 1.9 | 2.2 | 2.2 | 2.1 | 2.4 |  |
| Dadra and Nagar Haveli | 2.3 | 1.0 | 3.1 | 2.7 | 1.3 | 3.3 | 2.8 | 1.3 | 3.3 |  |
| Daman and Diu | 2.5 | 2.2 | 2.5 | 2.5 | 2.2 | 2.5 | 2.5 | 2.4 | 2.6 |  |
| Delhi | 2.3 | 1.7 | 2.8 | 2.4 | 1.8 | 3.0 | 3.5 | 3.1 | 3.9 |  |
| Goa | 4.0 | 3.0 | 5.0 | 4.2 | 3.2 | 5.2 | 5.7 | 4.8 | 6.3 |  |
| Gujarat | 1.6 | 1.4 | 1.8 | 1.8 | 1.6 | 2.0 | 2.3 | 2.1 | 2.4 |  |
| Haryana | 0.9 | 0.8 | 1.1 | 1.2 | 1.0 | 1.3 | 1.3 | 1.2 | 1.4 |  |
| Himachal Pradesh | 1.2 | 0.9 | 1.4 | 1.6 | 1.4 | 1.8 | 2.1 | 2.0 | 2.2 |  |
| Jammu and Kashmir | 1.2 | 0.9 | 1.6 | 1.7 | 1.4 | 2.0 | 2.1 | 1.8 | 2.4 |  |
| Jharkhand | 1.5 | 1.3 | 1.7 | 1.8 | 1.6 | 2.0 | 2.0 | 1.8 | 2.2 |  |
| Karnataka | 1.5 | 1.2 | 1.9 | 1.9 | 1.4 | 2.3 | 2.4 | 1.9 | 2.9 |  |
| Kerala | 2.2 | 1.8 | 2.6 | 3.2 | 2.8 | 3.6 | 4.4 | 4.0 | 4.7 |  |
| Lakshadweep | 2.4 | 1.3 | 3.6 | 3.0 | 1.8 | 4.1 | 4.4 | 3.4 | 4.9 |  |
| Madhya Pradesh | 1.2 | 1.1 | 1.3 | 1.4 | 1.3 | 1.5 | 1.7 | 1.6 | 1.8 |  |
| Maharashtra | 1.0 | 0.8 | 1.2 | 1.2 | 1.0 | 1.4 | 1.7 | 1.5 | 1.9 |  |
| Manipur | 1.6 | 1.4 | 1.8 | 1.9 | 1.6 | 2.1 | 2.3 | 2.1 | 2.4 |  |
| Meghalaya | 1.2 | 0.7 | 1.9 | 1.6 | 1.0 | 2.2 | 1.9 | 1.3 | 2.5 |  |
| Mizoram | 1.7 | 1.3 | 2.1 | 2.0 | 1.6 | 2.3 | 2.5 | 2.3 | 2.7 |  |
| Nagaland | 1.6 | 1.3 | 1.8 | 1.9 | 1.7 | 2.1 | 2.3 | 2.1 | 2.4 |  |
| Odisha | 2.1 | 1.9 | 2.3 | 2.7 | 2.5 | 2.9 | 3.3 | 3.1 | 3.4 |  |
| Puducherry | 2.1 | 1.4 | 2.7 | 2.5 | 1.8 | 3.0 | 3.2 | 2.6 | 3.7 |  |
| Punjab | 1.1 | 0.9 | 1.3 | 1.3 | 1.1 | 1.5 | 1.8 | 1.6 | 2.0 |  |
| Rajasthan | 0.8 | 0.7 | 0.9 | 1.0 | 0.9 | 1.1 | 1.2 | 1.1 | 1.3 |  |
| Sikkim | 1.0 | 0.7 | 1.4 | 1.2 | 0.9 | 1.5 | 2.0 | 1.7 | 2.2 |  |
| Tamil Nadu | 2.3 | 2.0 | 2.7 | 3.8 | 3.4 | 4.1 | 4.5 | 4.3 | 4.8 |  |
| Telangana | 2.0 | 1.5 | 2.4 | 2.3 | 1.9 | 2.7 | 3.2 | 2.9 | 3.5 |  |
| Tripura | 2.4 | 1.9 | 2.8 | 2.5 | 2.0 | 2.9 | 2.9 | 2.5 | 3.3 |  |
| Uttar Pradesh | 1.2 | 1.1 | 1.2 | 1.4 | 1.3 | 1.5 | 1.7 | 1.7 | 1.8 |  |
| Uttarakhand | 1.6 | 1.3 | 1.8 | 1.8 | 1.5 | 2.0 | 2.2 | 2.1 | 2.3 |  |
| West Bengal | 2.3 | 2.0 | 2.6 | 2.6 | 2.3 | 2.9 | 3.3 | 3.1 | 3.5 |  |

# Table S9. Total number of individuals with unmet need for care by state^a^

|  | **Unaware** | | | **Untreated** | | | **Uncontrolled** | | |
| --- | --- | --- | --- | --- | --- | --- | --- | --- | --- |
|  | **Estimate** | **Lower 95% CI** | **Upper 95% CI** | **Estimate** | **Lower 95% CI** | **Upper 95% CI** | **Estimate** | **Lower 95% CI** | **Upper 95% CI** |
| Andaman and Nicobar Islands | 8315 | 5755 | 11071 | 9307 | 6805 | 11844 | 13816 | 11900 | 15384 |
| Andhra Pradesh | 799979 | 676629 | 925282 | 908964 | 782805 | 1032315 | 1336226 | 1233467 | 1420897 |
| Arunachal Pradesh | 9800 | 7753 | 11782 | 12657 | 10860 | 14183 | 14036 | 12385 | 15336 |
| Assam | 216387 | 190042 | 242432 | 274612 | 247938 | 298992 | 329273 | 305838 | 348718 |
| Bihar | 683824 | 596575 | 772168 | 877460 | 785946 | 963954 | 1009852 | 913278 | 1095448 |
| Chandigarh | 10000 | 4636 | 16478 | 11231 | 5105 | 17972 | 18032 | 9326 | 22817 |
| Chhattisgarh | 282522 | 255975 | 304952 | 303569 | 280790 | 322084 | 326903 | 305644 | 342655 |
| Dadra and Nagar Haveli | 5006 | 2224 | 6684 | 5758 | 2761 | 6994 | 5969 | 2744 | 7087 |
| Daman and Diu | 4213 | 3843 | 4366 | 4221 | 3855 | 4370 | 4365 | 4126 | 4434 |
| Delhi | 237980 | 174497 | 299757 | 257618 | 193234 | 317314 | 374059 | 325644 | 406794 |
| Goa | 36512 | 27253 | 45275 | 38479 | 29326 | 46875 | 51943 | 44042 | 57605 |
| Gujarat | 585161 | 504110 | 662039 | 659155 | 580200 | 730405 | 814129 | 744008 | 868906 |
| Haryana | 136711 | 114605 | 158186 | 172190 | 152986 | 189215 | 191596 | 174815 | 205715 |
| Himachal Pradesh | 47712 | 38012 | 57681 | 65985 | 56410 | 74569 | 87462 | 81379 | 92040 |
| Jammu and Kashmir | 85964 | 64806 | 110260 | 121655 | 100125 | 143821 | 146550 | 123530 | 168807 |
| Jharkhand | 257953 | 220842 | 294571 | 315022 | 274284 | 352108 | 357102 | 313402 | 393605 |
| Karnataka | 566799 | 430684 | 717611 | 699852 | 536995 | 868496 | 897511 | 697899 | 1079327 |
| Kerala | 430222 | 351910 | 516408 | 635197 | 557766 | 712966 | 853942 | 788303 | 915153 |
| Lakshadweep | 960 | 525 | 1435 | 1195 | 726 | 1621 | 1729 | 1345 | 1955 |
| Madhya Pradesh | 482744 | 440433 | 524383 | 575414 | 538324 | 610626 | 687482 | 655490 | 716273 |
| Maharashtra | 668862 | 545343 | 797966 | 802828 | 680676 | 923408 | 1150608 | 1038365 | 1245009 |
| Manipur | 27299 | 23385 | 30965 | 31417 | 27348 | 34947 | 39214 | 36233 | 41381 |
| Meghalaya | 19235 | 11041 | 30022 | 24748 | 15973 | 34805 | 29361 | 20181 | 38949 |
| Mizoram | 11051 | 8555 | 13309 | 12717 | 10518 | 14546 | 16106 | 14709 | 17052 |
| Nagaland | 18530 | 15525 | 21260 | 22166 | 19281 | 24491 | 26062 | 23772 | 27587 |
| Odisha | 517279 | 465754 | 568909 | 648851 | 598253 | 697057 | 793353 | 742365 | 838155 |
| Puducherry | 16042 | 11125 | 21362 | 19061 | 14377 | 23696 | 25002 | 20389 | 28880 |
| Punjab | 179979 | 147648 | 214144 | 213702 | 178246 | 249403 | 303468 | 269193 | 333418 |
| Rajasthan | 297854 | 254960 | 340849 | 363172 | 323350 | 400810 | 457155 | 421744 | 487441 |
| Sikkim | 3971 | 2718 | 5354 | 4614 | 3369 | 5887 | 7698 | 6495 | 8519 |
| Tamil Nadu | 1039484 | 888052 | 1199337 | 1670035 | 1519130 | 1812278 | 2022309 | 1895293 | 2134317 |
| Telangana | 418576 | 328108 | 510428 | 495627 | 407781 | 578631 | 684990 | 610819 | 742227 |
| Tripura | 53751 | 43320 | 63283 | 56122 | 45681 | 65419 | 66001 | 55203 | 74385 |
| Uttar Pradesh | 1225532 | 1136322 | 1314045 | 1506638 | 1419466 | 1589832 | 1832003 | 1752498 | 1903258 |
| Uttarakhand | 90122 | 76960 | 102557 | 102375 | 88544 | 114610 | 127776 | 119705 | 134290 |
| West Bengal | 1284631 | 1119388 | 1442204 | 1448472 | 1286290 | 1596022 | 1838022 | 1732526 | 1924404 |

# Table S10. State-level prevalence estimates of ‘aware’, by sex

| Sex | State | Estimate | Lower 95% CI | Upper 95% CI |
| --- | --- | --- | --- | --- |
| Female | Andaman and Nicobar Islands | 71.9 | 62.5 | 79.6 |
| Female | Andhra Pradesh | 49.7 | 44.7 | 54.7 |
| Female | Arunachal Pradesh | 60.3 | 52.6 | 67.5 |
| Female | Assam | 51.3 | 46.4 | 56.2 |
| Female | Bihar | 61.3 | 55.7 | 66.7 |
| Female | Chandigarh | 77.4 | 54.1 | 90.9 |
| Female | Chhattisgarh | 30.9 | 25.8 | 36.6 |
| Female | Dadra and Nagar Haveli | 49.4 | 20.0 | 79.2 |
| Female | Daman and Diu | 31.6 | 13.5 | 57.9 |
| Female | Goa | 62.4 | 53.3 | 70.7 |
| Female | Gujarat | 69.6 | 56.9 | 80.0 |
| Female | Haryana | 50.6 | 44.7 | 56.5 |
| Female | Himachal Pradesh | 57.8 | 52.0 | 63.4 |
| Female | Jammu and Kashmir | 58.9 | 50.7 | 66.7 |
| Female | Jharkhand | 65.3 | 59.6 | 70.6 |
| Female | Karnataka | 39.3 | 34.3 | 44.6 |
| Female | Kerala | 63.6 | 53.8 | 72.4 |
| Female | Lakshadweep | 69.5 | 64.9 | 73.8 |
| Female | Madhya Pradesh | 69.8 | 55.0 | 81.4 |
| Female | Maharashtra | 55.4 | 51.6 | 59.1 |
| Female | Manipur | 58.4 | 53.0 | 63.6 |
| Female | Meghalaya | 40.5 | 34.3 | 47.0 |
| Female | Mizoram | 47.8 | 38.6 | 57.1 |
| Female | Nagaland | 44.5 | 36.0 | 53.4 |
| Female | Delhi | 41.2 | 33.2 | 49.8 |
| Female | Odisha | 49.3 | 45.3 | 53.3 |
| Female | Puducherry | 58.5 | 43.9 | 71.7 |
| Female | Punjab | 64.5 | 58.8 | 69.8 |
| Female | Rajasthan | 55.5 | 50.0 | 60.8 |
| Female | Sikkim | 68.9 | 58.1 | 78.0 |
| Female | Tamil Nadu | 67.9 | 64.4 | 71.2 |
| Female | Telangana | 57.4 | 49.3 | 65.2 |
| Female | Tripura | 48.2 | 38.2 | 58.3 |
| Female | Uttar Pradesh | 50.8 | 47.8 | 53.7 |
| Female | Uttarakhand | 57.3 | 50.0 | 64.3 |
| Female | West Bengal | 51.6 | 46.2 | 56.9 |
| Male | Andaman and Nicobar Islands | 44.1 | 20.8 | 70.2 |
| Male | Andhra Pradesh | 55.0 | 42.2 | 67.2 |
| Male | Arunachal Pradesh | 35.9 | 21.0 | 54.1 |
| Male | Assam | 44.5 | 33.7 | 55.8 |
| Male | Bihar | 43.0 | 33.6 | 52.9 |
| Male | Chandigarh | 46.6 | 3.2 | 95.8 |
| Male | Chhattisgarh | 22.1 | 14.0 | 33.2 |
| Male | Dadra and Nagar Haveli | 27.3 | 4.3 | 75.7 |
| Male | Daman and Diu | 0.0 | 0.0 | 0.0 |
| Male | Goa | 34.3 | 13.6 | 63.3 |
| Male | Gujarat | 39.1 | 23.0 | 58.0 |
| Male | Haryana | 37.7 | 27.3 | 49.3 |
| Male | Himachal Pradesh | 30.7 | 17.4 | 48.1 |
| Male | Jammu and Kashmir | 49.1 | 33.4 | 65.0 |
| Male | Jharkhand | 68.8 | 56.7 | 78.8 |
| Male | Karnataka | 52.0 | 41.3 | 62.4 |
| Male | Kerala | 61.8 | 45.2 | 76.1 |
| Male | Lakshadweep | 64.5 | 53.1 | 74.4 |
| Male | Madhya Pradesh | 38.7 | 8.5 | 81.0 |
| Male | Maharashtra | 36.7 | 29.0 | 45.1 |
| Male | Manipur | 54.8 | 38.2 | 70.4 |
| Male | Meghalaya | 40.3 | 26.7 | 55.6 |
| Male | Mizoram | 80.3 | 57.9 | 92.3 |
| Male | Nagaland | 36.2 | 14.5 | 65.4 |
| Male | Delhi | 35.6 | 20.8 | 53.7 |
| Male | Odisha | 51.3 | 43.6 | 59.0 |
| Male | Puducherry | 55.5 | 29.7 | 78.6 |
| Male | Punjab | 53.2 | 38.7 | 67.1 |
| Male | Rajasthan | 47.2 | 36.2 | 58.4 |
| Male | Sikkim | 49.1 | 25.0 | 73.6 |
| Male | Tamil Nadu | 55.7 | 45.0 | 65.9 |
| Male | Telangana | 46.4 | 28.6 | 65.2 |
| Male | Tripura | 35.7 | 21.3 | 53.3 |
| Male | Uttar Pradesh | 44.4 | 37.7 | 51.2 |
| Male | Uttarakhand | 24.8 | 12.0 | 44.4 |
| Male | West Bengal | 34.0 | 22.3 | 48.0 |

# Table S11. State-level prevalence estimates of ‘treated’, by sex

| Sex | State | Estimate | Lower 95% CI | Upper 95% CI |
| --- | --- | --- | --- | --- |
| Female | Andaman and Nicobar Islands | 64.7 | 54.9 | 73.3 |
| Female | Andhra Pradesh | 46.6 | 41.6 | 51.7 |
| Female | Arunachal Pradesh | 42.2 | 34.9 | 49.9 |
| Female | Assam | 38.9 | 34.0 | 44.0 |
| Female | Bihar | 45.8 | 39.9 | 51.8 |
| Female | Chandigarh | 65.6 | 41.4 | 83.7 |
| Female | Chhattisgarh | 26.5 | 21.6 | 32.1 |
| Female | Dadra and Nagar Haveli | 49.4 | 20.0 | 79.2 |
| Female | Daman and Diu | 30.6 | 12.8 | 57.0 |
| Female | Goa | 53.2 | 43.6 | 62.6 |
| Female | Gujarat | 68.4 | 55.7 | 78.8 |
| Female | Haryana | 40.0 | 34.4 | 45.8 |
| Female | Himachal Pradesh | 39.2 | 33.7 | 45.0 |
| Female | Jammu and Kashmir | 46.1 | 37.7 | 54.7 |
| Female | Jharkhand | 52.4 | 46.9 | 57.8 |
| Female | Karnataka | 34.7 | 29.9 | 39.9 |
| Female | Kerala | 53.6 | 42.2 | 64.7 |
| Female | Lakshadweep | 57.5 | 52.9 | 61.9 |
| Female | Madhya Pradesh | 49.2 | 32.0 | 66.6 |
| Female | Maharashtra | 43.1 | 39.5 | 46.8 |
| Female | Manipur | 46.6 | 41.4 | 52.0 |
| Female | Meghalaya | 35.3 | 29.3 | 41.7 |
| Female | Mizoram | 34.7 | 26.9 | 43.4 |
| Female | Nagaland | 41.1 | 32.7 | 50.0 |
| Female | Delhi | 27.0 | 21.0 | 34.0 |
| Female | Odisha | 38.2 | 34.6 | 41.8 |
| Female | Puducherry | 53.0 | 37.5 | 67.9 |
| Female | Punjab | 59.2 | 53.5 | 64.7 |
| Female | Rajasthan | 47.2 | 41.6 | 52.8 |
| Female | Sikkim | 60.2 | 49.9 | 69.7 |
| Female | Tamil Nadu | 53.3 | 49.6 | 56.9 |
| Female | Telangana | 51.4 | 43.3 | 59.5 |
| Female | Tripura | 40.9 | 31.8 | 50.6 |
| Female | Uttar Pradesh | 37.7 | 34.9 | 40.5 |
| Female | Uttarakhand | 43.3 | 36.6 | 50.4 |
| Female | West Bengal | 44.9 | 39.6 | 50.4 |
| Male | Andaman and Nicobar Islands | 40.5 | 18.7 | 66.9 |
| Male | Andhra Pradesh | 45.9 | 33.7 | 58.6 |
| Male | Arunachal Pradesh | 22.2 | 11.1 | 39.4 |
| Male | Assam | 29.0 | 19.7 | 40.4 |
| Male | Bihar | 31.8 | 23.1 | 42.0 |
| Male | Chandigarh | 46.6 | 3.2 | 95.8 |
| Male | Chhattisgarh | 15.9 | 9.6 | 25.3 |
| Male | Dadra and Nagar Haveli | 14.6 | 1.2 | 71.5 |
| Male | Daman and Diu | 0.0 | 0.0 | 0.0 |
| Male | Goa | 34.3 | 13.6 | 63.3 |
| Male | Gujarat | 35.8 | 20.5 | 54.5 |
| Male | Haryana | 31.8 | 22.0 | 43.6 |
| Male | Himachal Pradesh | 22.3 | 12.0 | 37.8 |
| Male | Jammu and Kashmir | 27.2 | 14.8 | 44.6 |
| Male | Jharkhand | 55.3 | 44.0 | 66.0 |
| Male | Karnataka | 37.0 | 26.2 | 49.2 |
| Male | Kerala | 54.3 | 36.9 | 70.7 |
| Male | Lakshadweep | 45.5 | 35.6 | 55.7 |
| Male | Madhya Pradesh | 38.7 | 8.5 | 81.0 |
| Male | Maharashtra | 28.1 | 21.8 | 35.3 |
| Male | Manipur | 49.4 | 33.7 | 65.1 |
| Male | Meghalaya | 27.8 | 15.2 | 45.2 |
| Male | Mizoram | 73.7 | 54.9 | 86.5 |
| Male | Nagaland | 21.2 | 7.0 | 49.2 |
| Male | Delhi | 25.2 | 12.4 | 44.3 |
| Male | Odisha | 37.8 | 30.7 | 45.4 |
| Male | Puducherry | 44.5 | 25.0 | 65.9 |
| Male | Punjab | 42.9 | 28.6 | 58.6 |
| Male | Rajasthan | 34.7 | 25.4 | 45.3 |
| Male | Sikkim | 44.4 | 21.4 | 70.1 |
| Male | Tamil Nadu | 25.2 | 18.1 | 33.9 |
| Male | Telangana | 34.8 | 19.6 | 53.8 |
| Male | Tripura | 35.7 | 21.3 | 53.3 |
| Male | Uttar Pradesh | 33.3 | 27.2 | 40.0 |
| Male | Uttarakhand | 23.4 | 10.9 | 43.3 |
| Male | West Bengal | 25.9 | 15.4 | 40.2 |

# Table S12. State-level prevalence estimates of ‘controlled’, by sex

| Sex | State | Estimate | Lower 95% CI | Upper 95% CI |
| --- | --- | --- | --- | --- |
| Female | Andaman and Nicobar Islands | 42.6 | 32.0 | 53.9 |
| Female | Andhra Pradesh | 23.8 | 19.7 | 28.5 |
| Female | Arunachal Pradesh | 31.5 | 24.8 | 39.1 |
| Female | Assam | 23.3 | 19.1 | 28.1 |
| Female | Bihar | 38.1 | 31.8 | 44.7 |
| Female | Chandigarh | 31.8 | 12.5 | 60.3 |
| Female | Chhattisgarh | 16.5 | 12.5 | 21.4 |
| Female | Dadra and Nagar Haveli | 33.9 | 9.7 | 71.0 |
| Female | Daman and Diu | 12.0 | 2.6 | 41.6 |
| Female | Goa | 28.4 | 20.9 | 37.3 |
| Female | Gujarat | 39.1 | 29.3 | 49.9 |
| Female | Haryana | 22.0 | 17.5 | 27.2 |
| Female | Himachal Pradesh | 30.0 | 25.1 | 35.4 |
| Female | Jammu and Kashmir | 22.3 | 16.9 | 28.8 |
| Female | Jharkhand | 38.1 | 32.2 | 44.4 |
| Female | Karnataka | 20.3 | 16.2 | 25.0 |
| Female | Kerala | 39.3 | 26.1 | 54.4 |
| Female | Lakshadweep | 37.5 | 33.0 | 42.1 |
| Female | Madhya Pradesh | 24.4 | 14.4 | 38.3 |
| Female | Maharashtra | 31.4 | 28.0 | 35.1 |
| Female | Manipur | 27.2 | 22.6 | 32.4 |
| Female | Meghalaya | 19.7 | 14.9 | 25.7 |
| Female | Mizoram | 30.6 | 22.7 | 39.9 |
| Female | Nagaland | 20.1 | 12.8 | 30.2 |
| Female | Delhi | 17.0 | 11.8 | 23.9 |
| Female | Odisha | 24.5 | 21.2 | 28.1 |
| Female | Puducherry | 31.8 | 19.6 | 47.1 |
| Female | Punjab | 30.4 | 25.5 | 35.7 |
| Female | Rajasthan | 33.1 | 27.7 | 38.9 |
| Female | Sikkim | 21.3 | 13.9 | 31.3 |
| Female | Tamil Nadu | 33.8 | 30.4 | 37.3 |
| Female | Telangana | 23.6 | 17.9 | 30.4 |
| Female | Tripura | 26.5 | 18.0 | 37.1 |
| Female | Uttar Pradesh | 23.8 | 21.2 | 26.5 |
| Female | Uttarakhand | 29.1 | 23.8 | 35.0 |
| Female | West Bengal | 27.1 | 22.8 | 31.9 |
| Male | Andaman and Nicobar Islands | 15.7 | 5.3 | 38.1 |
| Male | Andhra Pradesh | 18.7 | 11.0 | 29.9 |
| Male | Arunachal Pradesh | 17.0 | 7.7 | 33.7 |
| Male | Assam | 17.9 | 10.7 | 28.5 |
| Male | Bihar | 21.1 | 13.7 | 30.9 |
| Male | Chandigarh | 23.6 | 0.2 | 98.4 |
| Male | Chhattisgarh | 11.9 | 6.4 | 21.0 |
| Male | Dadra and Nagar Haveli | 14.6 | 1.2 | 71.5 |
| Male | Daman and Diu | 0.0 | 0.0 | 0.0 |
| Male | Goa | 7.9 | 1.0 | 43.0 |
| Male | Gujarat | 17.6 | 8.6 | 32.6 |
| Male | Haryana | 17.7 | 10.3 | 28.6 |
| Male | Himachal Pradesh | 16.6 | 8.1 | 31.0 |
| Male | Jammu and Kashmir | 8.7 | 3.3 | 21.0 |
| Male | Jharkhand | 48.0 | 36.9 | 59.4 |
| Male | Karnataka | 31.4 | 20.9 | 44.3 |
| Male | Kerala | 42.7 | 26.2 | 60.9 |
| Male | Lakshadweep | 31.2 | 23.8 | 39.6 |
| Male | Madhya Pradesh | 13.6 | 1.1 | 69.3 |
| Male | Maharashtra | 14.6 | 10.0 | 20.8 |
| Male | Manipur | 23.4 | 13.1 | 38.3 |
| Male | Meghalaya | 9.3 | 3.1 | 24.5 |
| Male | Mizoram | 64.8 | 46.2 | 79.9 |
| Male | Nagaland | 5.9 | 1.2 | 25.0 |
| Male | Delhi | 9.7 | 2.9 | 27.4 |
| Male | Odisha | 23.8 | 17.5 | 31.7 |
| Male | Puducherry | 34.4 | 17.3 | 56.8 |
| Male | Punjab | 30.6 | 18.1 | 46.8 |
| Male | Rajasthan | 18.0 | 11.4 | 27.4 |
| Male | Sikkim | 18.8 | 5.7 | 47.1 |
| Male | Tamil Nadu | 16.8 | 11.1 | 24.8 |
| Male | Telangana | 18.8 | 8.3 | 37.1 |
| Male | Tripura | 26.7 | 13.7 | 45.5 |
| Male | Uttar Pradesh | 19.3 | 14.3 | 25.5 |
| Male | Uttarakhand | 4.6 | 1.4 | 14.3 |
| Male | West Bengal | 8.6 | 3.7 | 18.6 |

# Table S13. State-level diabetes prevalence estimates, by sex

| **Sex** | **State** | **Estimate** | **Lower 95% CI** | **Upper 95% CI** |
| --- | --- | --- | --- | --- |
| Female | Andaman and Nicobar Islands | 6.6 | 5.3 | 8.2 |
| Female | Andhra Pradesh | 5.0 | 4.5 | 5.5 |
| Female | Arunachal Pradesh | 1.9 | 1.7 | 2.3 |
| Female | Assam | 2.1 | 1.9 | 2.4 |
| Female | Bihar | 2.7 | 2.3 | 3.0 |
| Female | Chandigarh | 3.3 | 2.3 | 4.8 |
| Female | Chhattisgarh | 1.9 | 1.6 | 2.1 |
| Female | Dadra and Nagar Haveli | 1.8 | 1.0 | 3.1 |
| Female | Daman and Diu | 1.8 | 1.0 | 3.2 |
| Female | Goa | 4.0 | 3.2 | 5.1 |
| Female | Gujarat | 5.6 | 4.6 | 6.9 |
| Female | Haryana | 2.5 | 2.2 | 2.8 |
| Female | Himachal Pradesh | 2.0 | 1.8 | 2.3 |
| Female | Jammu and Kashmir | 2.9 | 2.5 | 3.4 |
| Female | Jharkhand | 3.0 | 2.7 | 3.4 |
| Female | Karnataka | 1.8 | 1.6 | 2.0 |
| Female | Kerala | 4.2 | 3.3 | 5.2 |
| Female | Lakshadweep | 6.4 | 5.8 | 7.0 |
| Female | Madhya Pradesh | 5.6 | 4.3 | 7.2 |
| Female | Maharashtra | 2.2 | 2.0 | 2.3 |
| Female | Manipur | 2.5 | 2.2 | 2.8 |
| Female | Meghalaya | 2.4 | 2.1 | 2.8 |
| Female | Mizoram | 2.5 | 2.0 | 3.0 |
| Female | Nagaland | 2.9 | 2.3 | 3.6 |
| Female | Delhi | 2.1 | 1.8 | 2.5 |
| Female | Odisha | 3.1 | 2.9 | 3.4 |
| Female | Puducherry | 5.2 | 3.9 | 6.9 |
| Female | Punjab | 2.7 | 2.4 | 3.0 |
| Female | Rajasthan | 1.2 | 1.1 | 1.4 |
| Female | Sikkim | 2.4 | 1.9 | 2.9 |
| Female | Tamil Nadu | 5.6 | 5.2 | 6.0 |
| Female | Telangana | 4.0 | 3.4 | 4.8 |
| Female | Tripura | 3.1 | 2.6 | 3.8 |
| Female | Uttar Pradesh | 2.1 | 2.0 | 2.2 |
| Female | Uttarakhand | 2.6 | 2.3 | 2.9 |
| Female | West Bengal | 3.5 | 3.1 | 3.9 |
| Male | Andaman and Nicobar Islands | 8.9 | 6.0 | 13.2 |
| Male | Andhra Pradesh | 6.3 | 5.0 | 8.1 |
| Male | Arunachal Pradesh | 2.7 | 1.8 | 4.0 |
| Male | Assam | 2.5 | 1.9 | 3.2 |
| Male | Bihar | 2.9 | 2.4 | 3.5 |
| Male | Chandigarh | 3.8 | 1.2 | 11.4 |
| Male | Chhattisgarh | 3.3 | 2.6 | 4.2 |
| Male | Dadra and Nagar Haveli | 4.3 | 2.2 | 8.1 |
| Male | Daman and Diu | 2.9 | 1.2 | 6.6 |
| Male | Goa | 4.5 | 2.7 | 7.3 |
| Male | Gujarat | 7.8 | 5.5 | 11.0 |
| Male | Haryana | 3.0 | 2.4 | 3.7 |
| Male | Himachal Pradesh | 1.4 | 1.0 | 2.1 |
| Male | Jammu and Kashmir | 2.3 | 1.6 | 3.2 |
| Male | Jharkhand | 4.2 | 3.3 | 5.4 |
| Male | Karnataka | 3.8 | 3.1 | 4.8 |
| Male | Kerala | 4.1 | 2.9 | 5.7 |
| Male | Lakshadweep | 6.7 | 5.5 | 8.3 |
| Male | Madhya Pradesh | 5.3 | 2.9 | 9.5 |
| Male | Maharashtra | 2.2 | 1.9 | 2.6 |
| Male | Manipur | 2.2 | 1.6 | 2.9 |
| Male | Meghalaya | 2.9 | 2.1 | 4.1 |
| Male | Mizoram | 5.8 | 3.0 | 11.0 |
| Male | Nagaland | 3.0 | 1.9 | 4.6 |
| Male | Delhi | 3.1 | 2.2 | 4.5 |
| Male | Odisha | 5.7 | 4.8 | 6.6 |
| Male | Puducherry | 4.4 | 2.3 | 8.0 |
| Male | Punjab | 2.5 | 1.9 | 3.2 |
| Male | Rajasthan | 2.0 | 1.6 | 2.4 |
| Male | Sikkim | 2.6 | 1.6 | 4.2 |
| Male | Tamil Nadu | 6.5 | 5.2 | 8.0 |
| Male | Telangana | 4.2 | 3.0 | 5.8 |
| Male | Tripura | 4.6 | 3.1 | 6.8 |
| Male | Uttar Pradesh | 2.3 | 2.1 | 2.7 |
| Male | Uttarakhand | 2.8 | 2.0 | 3.8 |
| Male | West Bengal | 4.5 | 3.4 | 5.8 |

# Table S14. Multivariable logistic regressions of diabetes care indicators on socio-demographic characteristics without sampling weights^a^

|  | **Aware** | | | | **Treated** | | | | **Controlled** | | | |
| --- | --- | --- | --- | --- | --- | --- | --- | --- | --- | --- | --- | --- |
|  | **Rural** |  | **Urban** | | **Rural** | | **Urban** | | **Rural** | | **Urban** | |
|  | *OR (95% CI)* | *P* | *OR (95% CI)* | *P* | *OR (95% CI)* | *P* | *OR (95% CI)* | *P* | *OR (95% CI)* | *P* | *OR (95% CI)* | *P* |
| Education |  |  |  |  |  |  |  |  |  |  |  |  |
| Primary school or less | 1.00 (Ref.) |  | 1.00 (Ref.) |  | 1.00 (Ref.) |  | 1.00 (Ref.) |  | 1.00 (Ref.) |  | 1.00 (Ref.) |  |
| Secondary school unfinished | 1.26 (1.13-1.41) | <0.001 | 1.03 (0.91-1.18) | 0.623 | 1.31 (1.17-1.46) | <0.001 | 1.08 (0.95-1.23) | 0.252 | 1.14 (1.01-1.29) | 0.033 | 1.05 (0.91-1.22) | 0.5 |
| Secondary school or above | 1.28 (1.09-1.49) | 0.002 | 1.14 (0.97-1.34) | 0.124 | 1.29 (1.10-1.51) | 0.002 | 1.16 (0.98-1.37) | 0.076 | 1.28 (1.08-1.53) | 0.005 | 1.31 (1.10-1.57) | 0.003 |
| Household wealth quintile |  |  |  |  |  |  |  |  |  |  |  |  |
| Q1 (Poorest) | 1.00 (Ref.) |  | 1.00 (Ref.) |  | 1.00 (Ref.) |  | 1.00 (Ref.) |  | 1.00 (Ref.) |  | 1.00 (Ref.) |  |
| Q2 | 1.10 (0.92-1.31) | 0.308 | 1.11 (0.92-1.33) | 0.291 | 1.20 (0.99-1.45) | 0.067 | 1.24 (1.02-1.51) | 0.034 | 1.15 (0.95-1.41) | 0.158 | 1.18 (0.95-1.47) | 0.136 |
| Q3 | 1.20 (1.00-1.43) | 0.046 | 1.37 (1.14-1.67) | 0.001 | 1.36 (1.13-1.65) | 0.001 | 1.57 (1.28-1.92) | <0.001 | 1.19 (0.97-1.46) | 0.091 | 1.23 (0.98-1.53) | 0.069 |
| Q4 | 1.20 (1.00-1.44) | 0.049 | 1.43 (1.17-1.74) | <0.001 | 1.47 (1.21-1.79) | <0.001 | 1.67 (1.37-2.05) | <0.001 | 1.07 (0.87-1.32) | 0.503 | 1.18 (0.94-1.48) | 0.156 |
| Q5 (Richest) | 1.48 (1.23-1.79) | <0.001 | 1.86 (1.50-2.30) | <0.001 | 2.05 (1.68-2.50) | <0.001 | 2.22 (1.78-2.76) | <0.001 | 1.33 (1.07-1.64) | 0.01 | 1.55 (1.22-1.96) | <0.001 |
| Currently married | 1.06 (0.93-1.22) | 0.399 | 1.19 (1.01-1.39) | 0.036 | 1.01 (0.88-1.16) | 0.899 | 1.26 (1.07-1.48) | 0.006 | 1.11 (0.95-1.30) | 0.187 | 1.08 (0.91-1.30) | 0.378 |
| Female | 1.28 (1.12-1.46) | <0.001 | 1.72 (1.47-2.02) | <0.001 | 1.44 (1.26-1.66) | <0.001 | 1.99 (1.68-2.34) | <0.001 | 1.29 (1.11-1.50) | 0.001 | 2.12 (1.75-2.57) | <0.001 |

Abbreviations: OR = Odds Ratio; CI = Confidence Interval; Ref. = Reference group; Q = Quintile

a These regressions contained all sociodemographic variables listed in the table (wealth quintile, education, marital status, and sex), age as a continuous variable with restricted cubic splines with five knots (the knots were placed at the fifth, 27.5^th^, 50^th^, 72.5^th^, and 95^th^ percentiles), and a binary indicator for each district (district-level fixed effects) as explanatory variables. Standard errors were adjusted for clustering at the primary sampling unit (PSU) level.

# Table S15. Multivariable (weighted) logistic regressions of diabetes care indicators on socio-demographic characteristics, with an interaction term between household wealth quintile and rural/urban location^a^

|  | **Aware** | | **Treated** | | **Controlled** | |
| --- | --- | --- | --- | --- | --- | --- |
|  | *OR (95% CI)* | *P* | *OR (95% CI)* | *P* | *OR (95% CI)* | *P* |
| Education |  |  |  |  |  |  |
| Primary school or less | 1.00 (Ref.) |  | 1.00 (Ref.) |  | 1.00 (Ref.) |  |
| Secondary school unfinished | 1.17 (1.11-1.24) | <0.001 | 1.33 (1.26-1.41) | <0.001 | 1.00 (0.93-1.06) | 0.919 |
| Secondary school or above | 1.47 (1.37-1.57) | <0.001 | 1.43 (1.33-1.54) | <0.001 | 1.39 (1.29-1.51) | <0.001 |
| Household wealth quintile |  |  |  |  |  |  |
| Q1 (Poorest) | 1.00 (Ref.) |  | 1.00 (Ref.) |  | 1.00 (Ref.) |  |
| Q2 | 0.81 (0.71-0.91) | 0.001 | 0.91 (0.79-1.03) | 0.145 | 1.04 (0.90-1.20) | 0.593 |
| Q3 | 0.89 (0.78-1.00) | 0.056 | 0.99 (0.87-1.13) | 0.911 | 1.07 (0.92-1.23) | 0.38 |
| Q4 | 0.95 (0.84-1.07) | 0.387 | 1.22 (1.08-1.39) | 0.002 | 0.96 (0.83-1.10) | 0.544 |
| Q5 (Richest) | 1.02 (0.90-1.15) | 0.735 | 1.42 (1.25-1.61) | <0.001 | 1.07 (0.93-1.23) | 0.359 |
| Currently married | 1.00 (0.93-1.07) | 0.945 | 1.16 (1.07-1.25) | <0.001 | 1.34 (1.23-1.46) | <0.001 |
| Female | 1.61 (1.53-1.68) | <0.001 | 1.78 (1.70-1.87) | <0.001 | 1.71 (1.62-1.80) | <0.001 |
| Urban | 0.94 (0.82-1.08) | 0.361 | 0.97 (0.84-1.12) | 0.695 | 0.79 (0.67-0.93) | 0.005 |
| Q2:Urban | 1.41 (1.19-1.66) | <0.001 | 1.43 (1.20-1.72) | <0.001 | 1.00 (0.82-1.23) | 0.998 |
| Q3:Urban | 1.45 (1.23-1.71) | <0.001 | 1.96 (1.65-2.34) | <0.001 | 1.45 (1.20-1.77) | <0.001 |
| Q4:Urban | 1.12 (0.96-1.32) | 0.155 | 1.32 (1.11-1.56) | 0.002 | 1.40 (1.16-1.70) | 0.001 |
| Q5:Urban | 1.61 (1.37-1.89) | <0.001 | 1.74 (1.47-2.06) | <0.001 | 1.58 (1.30-1.90) | <0.001 |

a These regressions contained all sociodemographic variables listed in the table (wealth quintile, education, marital status, urban/rural and sex), age as a continuous variable with restricted cubic splines with five knots (the knots were placed at the fifth, 27.5^th^, 50^th^, 72.5^th^, and 95^th^ percentiles), an interaction term for household wealth quintile and rural/urban location and a binary indicator for each district (district-level fixed effects) as explanatory variables.

# Table S16. Multivariable (weighted) logistic regressions of diabetes care indicators on socio-demographic characteristics, with an interaction term between education and rural/urban location^a^

|  | **Aware** | | **Treated** | | **Controlled** | |
| --- | --- | --- | --- | --- | --- | --- |
|  | *OR (95% CI)* | *P* | *OR (95% CI)* | *P* | *OR (95% CI)* | *P* |
| Education |  |  |  |  |  |  |
| Primary school or less | 1.00 (Ref.) |  | 1.00 (Ref.) |  | 1.00 (Ref.) |  |
| Secondary school unfinished | 1.24 (1.16-1.33) | <0.001 | 1.39 (1.30-1.50) | <0.001 | 1.02 (0.94-1.10) | 0.691 |
| Secondary school or above | 1.34 (1.22-1.47) | <0.001 | 1.32 (1.20-1.45) | <0.001 | 1.13 (1.02-1.26) | 0.023 |
| Household wealth quintile |  |  |  |  |  |  |
| Q1 (Poorest) | 1.00 (Ref.) |  | 1.00 (Ref.) |  | 1.00 (Ref.) |  |
| Q2 | 0.97 (0.89-1.06) | 0.536 | 1.10 (1.01-1.21) | 0.037 | 1.03 (0.93-1.14) | 0.584 |
| Q3 | 1.08 (0.99-1.18) | 0.067 | 1.44 (1.32-1.58) | <0.001 | 1.29 (1.17-1.43) | <0.001 |
| Q4 | 1.02 (0.94-1.11) | 0.602 | 1.44 (1.31-1.57) | <0.001 | 1.13 (1.02-1.25) | 0.018 |
| Q5 (Richest) | 1.27 (1.17-1.39) | <0.001 | 1.87 (1.70-2.06) | <0.001 | 1.32 (1.19-1.47) | <0.001 |
| Currently married | 1.00 (0.93-1.08) | 0.985 | 1.16 (1.07-1.25) | <0.001 | 1.34 (1.23-1.46) | <0.001 |
| Female | 1.60 (1.53-1.68) | <0.001 | 1.78 (1.69-1.86) | <0.001 | 1.70 (1.62-1.79) | <0.001 |
| Urban | 1.29 (1.18-1.40) | <0.001 | 1.49 (1.37-1.63) | <0.001 | 0.98 (0.89-1.08) | 0.733 |
| Secondary school unfinished:Urban | 0.87 (0.79-0.97) | 0.009 | 0.90 (0.81-1.00) | 0.049 | 0.96 (0.85-1.09) | 0.543 |
| Secondary school or above:Urban | 1.16 (1.03-1.31) | 0.016 | 1.16 (1.02-1.31) | 0.02 | 1.46 (1.27-1.67) | <0.001 |

a These regressions contained all sociodemographic variables listed in the table (wealth quintile, education, marital status, urban/rural and sex), age as a continuous variable with restricted cubic splines with five knots (the knots were placed at the fifth, 27.5^th^, 50^th^, 72.5^th^, and 95^th^ percentiles), an interaction term for education and rural/urban location and a binary indicator for each district (district-level fixed effects) as explanatory variables.

# Table S17. Multivariable logistic regressions of diabetes care indicators on socio-demographic characteristics, among women only^a^

|  | **Aware** | | | | **Treated** | | | | **Controlled** | | | |
| --- | --- | --- | --- | --- | --- | --- | --- | --- | --- | --- | --- | --- |
|  | **Rural** |  | **Urban** | | **Rural** | | **Urban** | | **Rural** | | **Urban** | |
|  | *OR (95% CI)* | *P* | *OR (95% CI)* | *P* | *OR (95% CI)* | *P* | *OR (95% CI)* | *P* | *OR (95% CI)* | *P* | *OR (95% CI)* | *P* |
| Education |  |  |  |  |  |  |  |  |  |  |  |  |
| Primary school or less | 1.00 (Ref.) |  | 1.00 (Ref.) |  | 1.00 (Ref.) |  | 1.00 (Ref.) |  | 1.00 (Ref.) |  | 1.00 (Ref.) |  |
| Secondary school unfinished | 1.31 (1.16-1.48) | <0.001 | 1.04 (0.90-1.21) | 0.576 | 1.31 (1.16-1.49) | <0.001 | 1.04 (0.90-1.20) | 0.582 | 1.17 (1.03-1.34) | 0.019 | 1.01 (0.87-1.19) | 0.872 |
| Secondary school or above | 1.28 (1.07-1.54) | 0.008 | 1.16 (0.97-1.39) | 0.112 | 1.20 (0.99-1.44) | 0.059 | 1.14 (0.95-1.37) | 0.146 | 1.26 (1.03-1.54) | 0.023 | 1.26 (1.04-1.52) | 0.02 |
| Household wealth quintile |  |  |  |  |  |  |  |  |  |  |  |  |
| Q1 (Poorest) | 1.00 (Ref.) |  | 1.00 (Ref.) |  | 1.00 (Ref.) |  | 1.00 (Ref.) |  | 1.00 (Ref.) |  | 1.00 (Ref.) |  |
| Q2 | 1.11 (0.91-1.36) | 0.299 | 1.13 (0.92-1.39) | 0.251 | 1.29 (1.03-1.60) | 0.023 | 1.27 (1.02-1.58) | 0.032 | 1.22 (0.97-1.52) | 0.086 | 1.28 (1.01-1.61) | 0.04 |
| Q3 | 1.29 (1.06-1.58) | 0.011 | 1.40 (1.13-1.72) | 0.002 | 1.52 (1.23-1.88) | <0.001 | 1.56 (1.25-1.94) | <0.001 | 1.26 (1.01-1.58) | 0.043 | 1.29 (1.02-1.64) | 0.035 |
| Q4 | 1.24 (1.01-1.52) | 0.038 | 1.46 (1.17-1.81) | 0.001 | 1.59 (1.27-1.97) | <0.001 | 1.73 (1.39-2.16) | <0.001 | 1.17 (0.93-1.48) | 0.178 | 1.25 (0.98-1.59) | 0.075 |
| Q5 (Richest) | 1.56 (1.26-1.93) | <0.001 | 1.81 (1.42-2.29) | <0.001 | 2.29 (1.83-2.87) | <0.001 | 2.17 (1.70-2.77) | <0.001 | 1.42 (1.12-1.80) | 0.004 | 1.66 (1.28-2.16) | <0.001 |
| Currently married | 1.09 (0.94-1.27) | 0.252 | 1.22 (1.02-1.46) | 0.026 | 1.03 (0.88-1.20) | 0.748 | 1.20 (1.01-1.44) | 0.04 | 1.16 (0.97-1.38) | 0.094 | 1.06 (0.87-1.29) | 0.567 |

Abbreviations: OR = Odds Ratio; CI = Confidence Interval; Q = Quintile; P= P-value for OR

a These regressions contained all sociodemographic variables listed in the table (wealth quintile, education, marital status, and sex), age as a continuous variable with restricted cubic splines with five knots (the knots were placed at the fifth, 27.5^th^, 50^th^, 72.5^th^, and 95^th^ percentiles), and a binary indicator for each district (district-level fixed effects) as explanatory variables. Standard errors were adjusted for clustering at the primary sampling unit (PSU) level.

# Table S18. Multivariable logistic regressions of diabetes care indicators on socio-demographic characteristics, among men only^a^

|  | **Aware** | | | | **Treated** | | | | **Controlled** | | | |
| --- | --- | --- | --- | --- | --- | --- | --- | --- | --- | --- | --- | --- |
|  | **Rural** |  | **Urban** | | **Rural** | | **Urban** | | **Rural** | | **Urban** | |
|  | *OR (95% CI)* | *P* | *OR (95% CI)* | *P* | *OR (95% CI)* | *P* | *OR (95% CI)* | *P* | *OR (95% CI)* | *P* | *OR (95% CI)* | *P* |
| Education |  |  |  |  |  |  |  |  |  |  |  |  |
| Primary school or less | 1.00 (Ref.) |  | 1.00 (Ref.) |  | 1.00 (Ref.) |  | 1.00 (Ref.) |  | 1.00 (Ref.) |  | 1.00 (Ref.) |  |
| Secondary school unfinished | 1.37 (0.90-2.07) | 0.14 | 1.29 (0.66-2.52) | 0.449 | 1.83 (1.17-2.85) | 0.008 | 1.65 (0.82-3.33) | 0.162 | 1.33 (0.81-2.19) | 0.265 | 1.57 (0.65-3.81) | 0.32 |
| Secondary school or above | 1.52 (0.92-2.53) | 0.106 | 1.24 (0.61-2.54) | 0.557 | 2.06 (1.20-3.53) | 0.008 | 1.52 (0.71-3.22) | 0.28 | 1.64 (0.89-2.99) | 0.111 | 2.69 (1.09-6.62) | 0.032 |
| Household wealth quintile |  |  |  |  |  |  |  |  |  |  |  |  |
| Q1 (Poorest) | 1.00 (Ref.) |  | 1.00 (Ref.) |  | 1.00 (Ref.) |  | 1.00 (Ref.) |  | 1.00 (Ref.) |  | 1.00 (Ref.) |  |
| Q2 | 1.00 (0.52-1.93) | 0.996 | 0.66 (0.29-1.50) | 0.319 | 0.81 (0.41-1.62) | 0.552 | 0.83 (0.33-2.10) | 0.699 | 0.92 (0.45-1.86) | 0.809 | 0.32 (0.11-0.94) | 0.037 |
| Q3 | 0.79 (0.40-1.55) | 0.495 | 0.81 (0.35-1.85) | 0.615 | 0.66 (0.33-1.34) | 0.248 | 1.25 (0.49-3.19) | 0.635 | 0.88 (0.43-1.83) | 0.735 | 0.45 (0.15-1.31) | 0.142 |
| Q4 | 1.23 (0.62-2.46) | 0.556 | 0.90 (0.38-2.17) | 0.818 | 1.25 (0.62-2.54) | 0.528 | 1.18 (0.45-3.08) | 0.732 | 0.82 (0.38-1.75) | 0.61 | 0.48 (0.17-1.35) | 0.165 |
| Q5 (Richest) | 1.37 (0.66-2.84) | 0.401 | 1.34 (0.51-3.53) | 0.556 | 1.61 (0.76-3.43) | 0.215 | 2.19 (0.77-6.20) | 0.141 | 1.27 (0.57-2.84) | 0.565 | 0.49 (0.16-1.54) | 0.223 |
| Currently married | 0.78 (0.44-1.39) | 0.405 | 0.90 (0.43-1.85) | 0.767 | 0.85 (0.48-1.51) | 0.581 | 1.54 (0.68-3.46) | 0.3 | 1.13 (0.61-2.09) | 0.69 | 1.39 (0.45-4.30) | 0.567 |

Abbreviations: OR = Odds Ratio; CI = Confidence Interval; Q = Quintile; P= P-value for OR

a These regressions contained all sociodemographic variables listed in the table (wealth quintile, education, marital status, and sex), age as a continuous variable with restricted cubic splines with five knots (the knots were placed at the fifth, 27.5^th^, 50^th^, 72.5^th^, and 95^th^ percentiles), and a binary indicator for each district (district-level fixed effects) as explanatory variables. Standard errors were adjusted for clustering at the primary sampling unit (PSU) level.

# Figure S1. Map of diabetes prevalence by state, age group 15 - 49 years


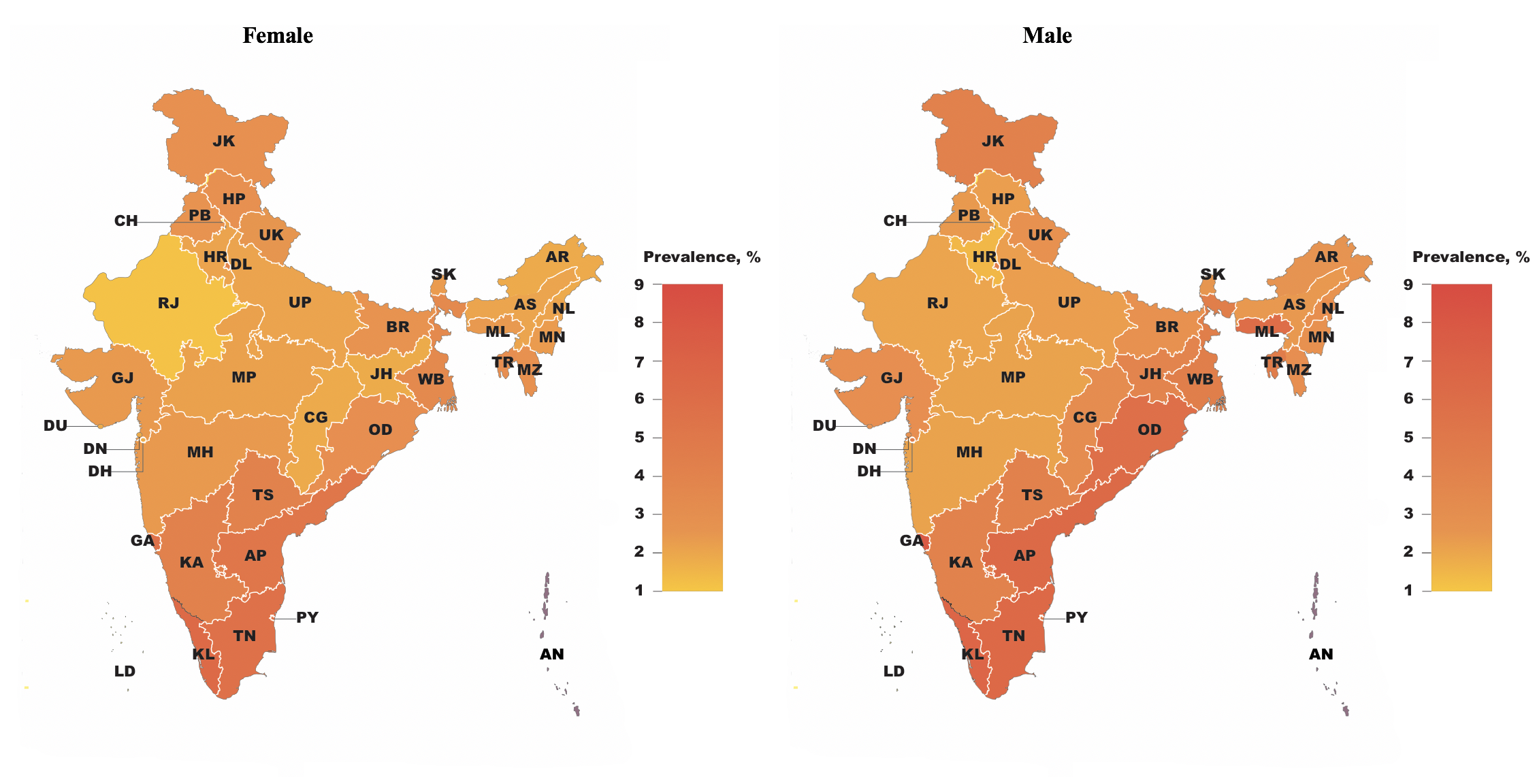


^a^ Point estimates with 95% confidence intervals can be found in Table S13.

# Figure S2. The association between district-level diabetes prevalence and care cascade indicators, separately for age group 15 - 29 years, 30 - 39 years, and 40 - 49 years ^a,b,c^


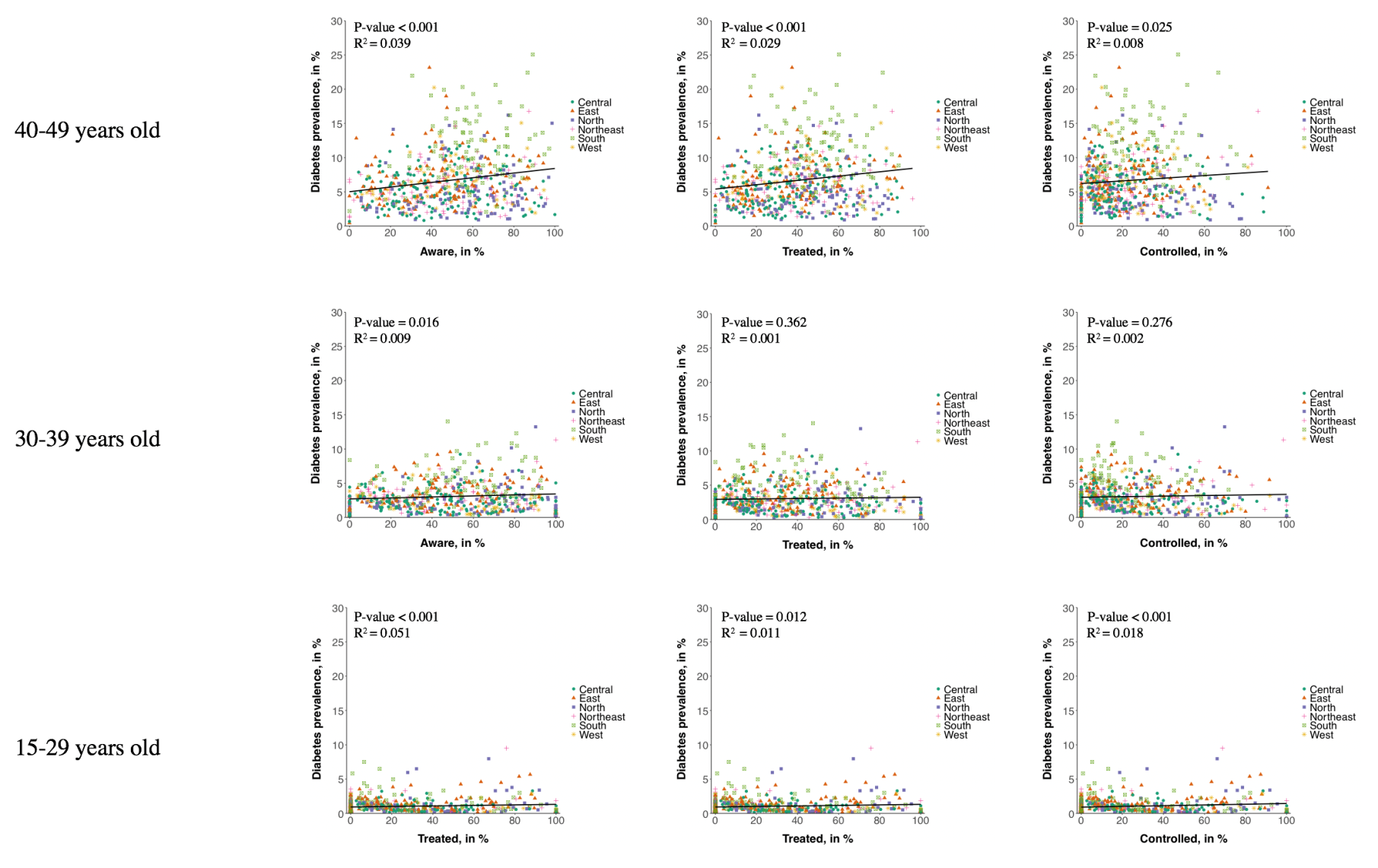


^a^ All estimates were age-standardized to the Global Burden of Disease Project’s age structure for India for 2015.[[11](#_ENREF_11)]

^b^ P-values indicate the statistical significance of the slope of the regression line shown in black, which is an ordinary least squares regression of district-level diabetes prevalence onto district-level awareness among those with diabetes. R^2^ values are for the same regression.

^c^  19,453 individuals with diabetes were included in this figure; 10,504 were 'aware', 8,269 'treated', and 5,329 'controlled'.

# Figure S3. The association between state-level diabetes prevalence and care cascade indicators^a,b,c,d^

**
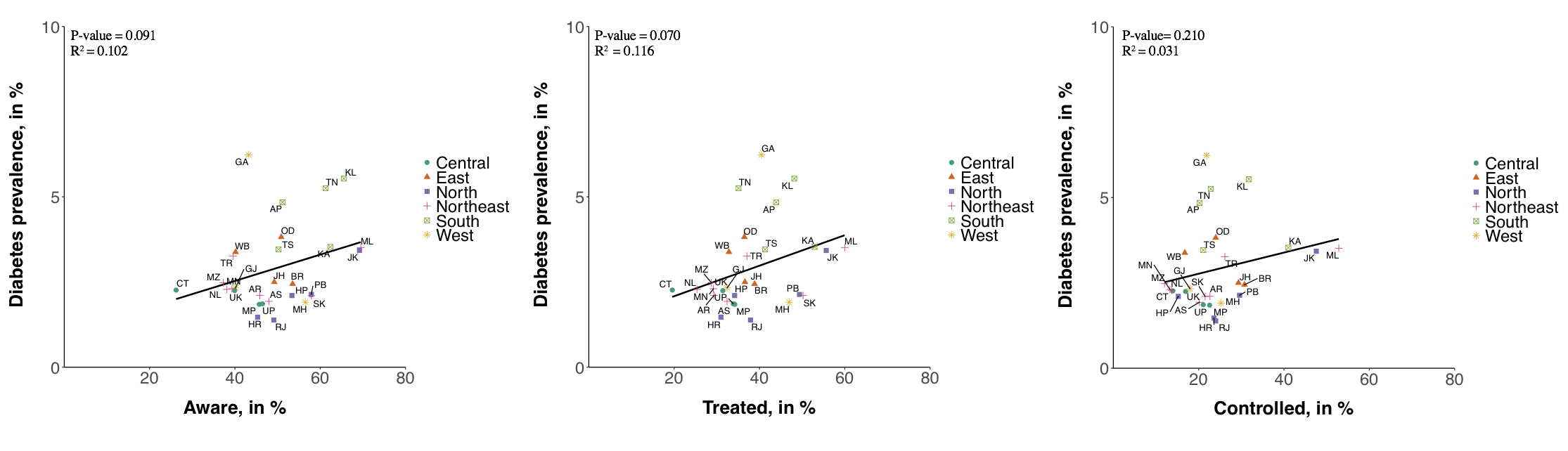
**

^a^ All estimates were age-standardized to the Global Burden of Disease Project’s age structure for India for 2015.[[11](#_ENREF_11)]

^b^ P-values indicate the statistical significance of the slope of the regression line shown in black, which is an ordinary least squares regression of state-level diabetes prevalence onto state-level awareness among those with diabetes. R^2^ values are for the same regression.

^c^ AP indicates Andhra Pradesh; AR, Arunachal Pradesh; AS, Assam; BR, Bihar; CT, Chhattisgarh; GA, Goa; GR, Gujarat; HR, Haryana; HP, Himachal Pradesh, JK, Jammu and Kashmir; JH, Jharkhand; KA, Karnataka; KL, Kerala; MP, Madhya Pradesh; MH, Maharashtra; MN, Manipur; ML, Meghalaya; MZ, Mizoram; NL, Nagaland; OD, Odisha (Orissa); PB, Punjab; RJ, Rajasthan; SK, Sikkim; TN, Tamil Nadu; TS, Telangana State; TR, Tripura; UP, Uttar Pradesh; UK, Uttarakhand (Uttaranchal); WB, West Bengal.

^d^  19,453 individuals with diabetes were included in this figure; 10,504 were 'aware', 8,269 'treated', and 5,329 'controlled'.

# Figure S4. Predicted probabilities of awareness, treatment, and control by age group, rural or urban location, and household wealth quintile^a^


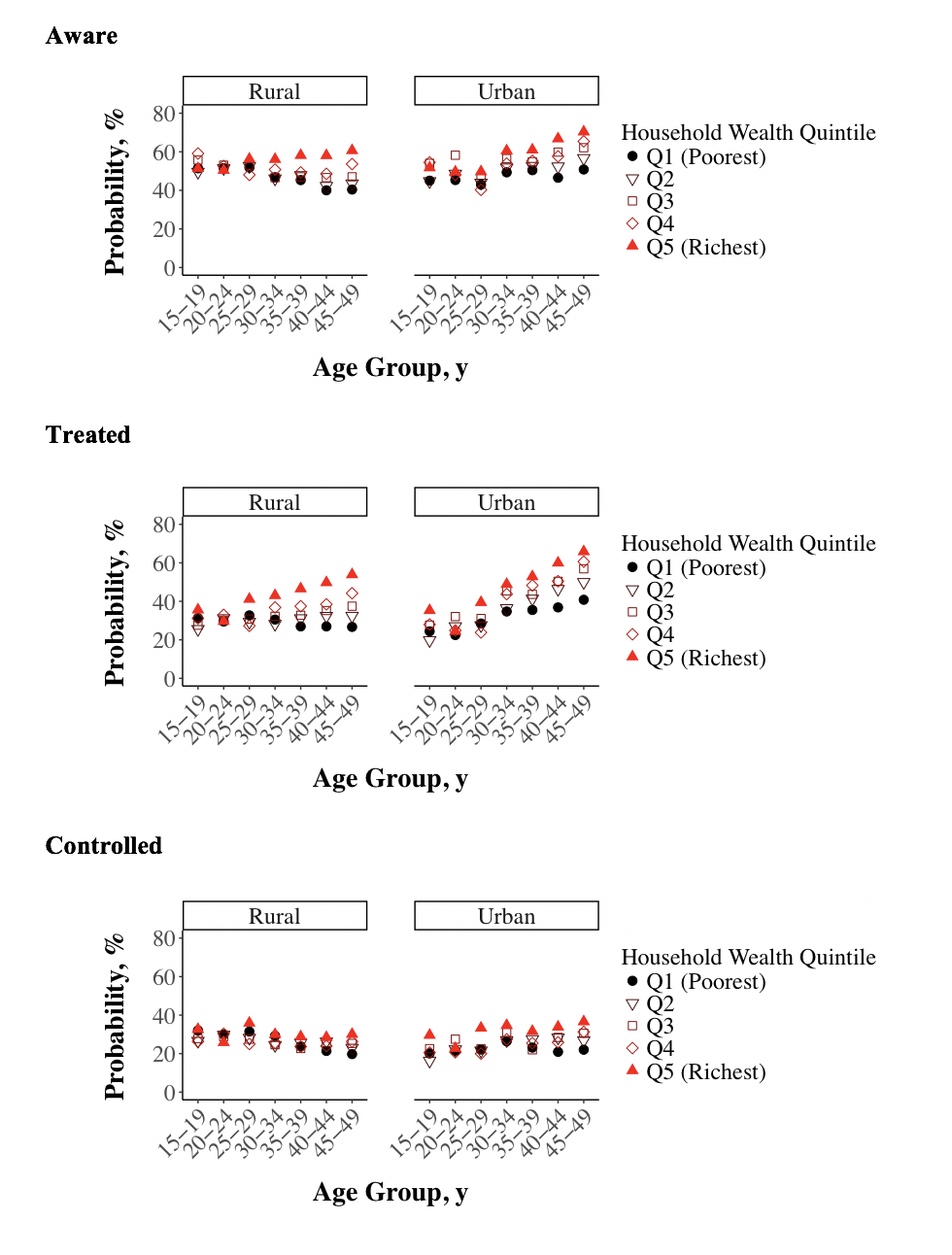


^a^ Predicted probabilities were obtained from multivariable logistic regressions of diabetes care indicators on individuals’ sociodemographic characteristics (age group, household wealth quintile, education, marital status, sex, and rural vs. urban location), district-level fixed effects, and an interaction term between i) age group and household wealth quintile, ii) age group and rural/urban, and iii) household wealth quintile and rural/urban.

# Figure S5. Flowchart of the proportion of patients lost in the diabetes care cascade


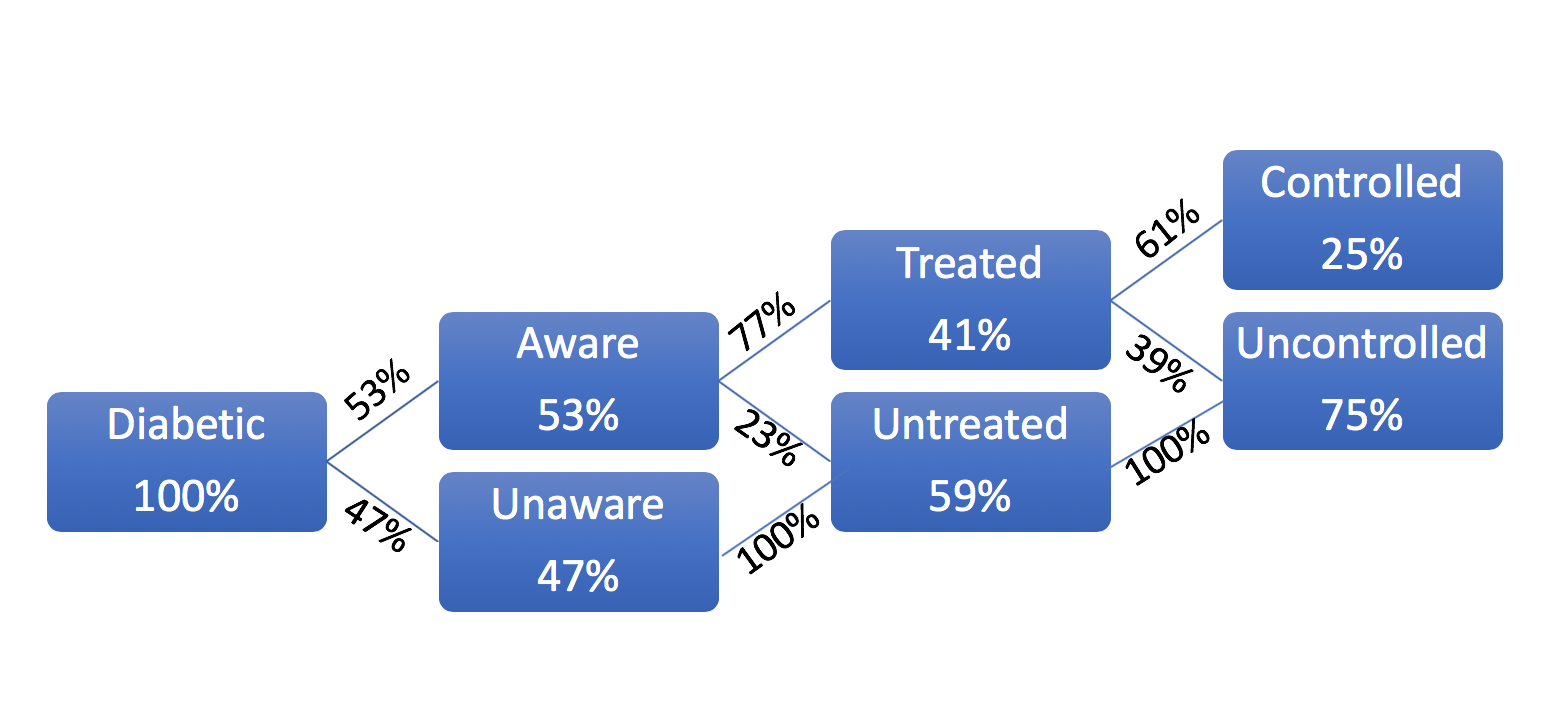


# References

1. International Diabetes Federation: **Recommendations for Managing Type 2 Diabetes in Primary Care**. 2017. [www.idf.org/managing-type2-diabetes](file:///Users/jonasprenissl/Documents/Public%20Health%20Files/Public%20Health/Paper/Submitted%20versions/PLOS%20Medicine%20Hypertension%20cascades/Revise%20and%20Resubmit/diabetes/www.idf.org/managing-type2-diabetes).

2. International Institute for Population Sciences Mumbai: **National Family Health Survey 2015-2016 (NFHS-4) Clinical Anthropometric Biochemical (CAB) Manual,** [**http://rchiips.org/NFHS/NFHS4/manual/NFHS-4%20Biomarker%20Field%20Manual.pdf**](http://rchiips.org/NFHS/NFHS4/manual/NFHS-4%20Biomarker%20Field%20Manual.pdf). 2014.

3. International Institute for Population Sciences Mumbai: **National Family Health Survey 2015-2016 (NFHS-4) Interviewer Manual,** [**http://rchiips.org/NFHS/NFHS4/manual/NFHS-4%20Interviewer%20Manual.pdf**](http://rchiips.org/NFHS/NFHS4/manual/NFHS-4%20Interviewer%20Manual.pdf)**.**

. 2014.

4. International Institute for Population Sciences Mumbai: **National Family Health Survey 2015-2016 (NFHS-4) Supervisor Manual,** [**http://rchiips.org/NFHS/NFHS4/manual/NFHS-4%20Supervisor%20Manual.pdf**](http://rchiips.org/NFHS/NFHS4/manual/NFHS-4%20Supervisor%20Manual.pdf).

5. International Institute for Population Sciences Mumbai: **International Institute for Population Sciences Mumbai. National Family Health Survey 2015-2016 (NFHS-4) Man’s Questionnaire,** [**http://rchiips.org/NFHS/NFHS4/schedules/NFHS-4Mans.pdf**](http://rchiips.org/NFHS/NFHS4/schedules/NFHS-4Mans.pdf).

6. International Institute for Population Sciences Mumbai: **National Family Health Survey 2015-2016 (NFHS-4) Biomarker Questionnaire,** [**http://rchiips.org/NFHS/NFHS4/schedules/NFHS-4Biomarker.pdf**](http://rchiips.org/NFHS/NFHS4/schedules/NFHS-4Biomarker.pdf). 2014.

7. International Institute for Population Sciences Mumbai: **National Family Health Survey 2015-2016 (NFHS-4) Household Questionnaire,** [**http://rchiips.org/NFHS/NFHS4/schedules/NFHS-4Household.pdf**](http://rchiips.org/NFHS/NFHS4/schedules/NFHS-4Household.pdf). 2014.

8. International Institute for Population Sciences Mumbai: **International Institute for Population Sciences Mumbai. National Family Health Survey 2015-2016 (NFHS-4) Woman’s Questionnaire,** [**http://rchiips.org/NFHS/NFHS4/schedules/NFHS-4Womans.pdf**](http://rchiips.org/NFHS/NFHS4/schedules/NFHS-4Womans.pdf).

9. International Institute for Population Sciences Mumbai: **National Family Health Survey (NFHS-4), 2015-16: India Report, available from** [**http://rchiips.org/nfhs/NFHS-4Reports/India.pdf**](http://rchiips.org/nfhs/NFHS-4Reports/India.pdf) 2017.

10. Institute for Health Metrics and Evaluation: **GBD Compare online visualization hub. Seattle, WA: IHME, University of Washington**. *Available from* [*http://vizhubhealthdataorg/gbd-compare*](http://vizhubhealthdataorg/gbd-compare) *(Accessed [08-31-2018])* 2015

11. Global Burden of Disease Collaborative Network: **Global Burden of Disease Study 2015 (GBD 2015) Population Estimates 1970-2015**. *Seattle, United States: Institute for Health Metrics and Evaluation (IHME)* 2016.
